# Efficacy of Polyphenols in the Management of Dyslipidemia: A Focus on Clinical Studies

**DOI:** 10.3390/nu13020672

**Published:** 2021-02-19

**Authors:** Francis Feldman, Mireille Koudoufio, Yves Desjardins, Schohraya Spahis, Edgard Delvin, Emile Levy

**Affiliations:** 1 Research Centre, Sainte-Justine University Health Center, Montreal, QC H3T 1C5, Canada; francis.feldman@umontreal.ca (F.F.); mireille.koudoufio@umontreal.ca (M.K.); schohraya.spahis.hsj@ssss.gouv.qc.ca (S.S.); delvine@sympatico.ca (E.D.); 2 Department of Nutrition, Montreal University, Montreal, QC H3T 1A8, Canada; 3 Institute of Nutrition and Functional Foods, Laval University, Quebec, QC G1V 4L3, Canada; Yves.Desjardins@fsaa.ulaval.ca; 4 Department of Biochemistry, Montreal University, Montreal, QC H3T 1J4, Canada

**Keywords:** polyphenols, dyslipidemia, lipoproteins, nutrition, oxidative stress, inflammation, microbiota, metabolic syndrome, type 2 diabetes

## Abstract

Polyphenols (PLPs), phytochemicals found in a wide range of plant-based foods, have gained extensive attention in view of their antioxidant, anti-inflammatory, immunomodulatory and several additional beneficial activities. The health-promoting effects noted in animal models of various non-communicable diseases explain the growing interest in these molecules. In particular, in vitro and animal studies reported an attenuation of lipid disorders in response to PLPs. However, despite promising preclinical investigations, the effectiveness of PLPs in human dyslipidemia (DLP) is less clear and necessitates revision of available literature. Therefore, the present review analyzes the role of PLPs in managing clinical DLP, notably by dissecting their potential in ameliorating lipid/lipoprotein metabolism and alleviating hyperlipidemia, both postprandially and in long-term interventions. To this end, PubMed was used for article search. The search terms included polyphenols, lipids, triglycerides, cholesterol, LDL-cholesterol and /or HDL-cholesterol. The critical examination of the trials published to date illustrates certain benefits on blood lipids along with co-morbidities in participant’s health status. However, inconsistent results document significant research gaps, potentially owing to study heterogeneity and lack of rigor in establishing PLP bioavailability during supplementation. This underlines the need for further efforts in order to elucidate and support a potential role of PLPs in fighting DLP.

## 1. Introduction

Cardiovascular disease (CVD) is one of the leading causes of morbidity and mortality in the world. It represents a major concern for global health, and its prevalence as of 2017 is estimated to be around 423 million cases with 18 million deaths [1]. Not surprisingly, its crippling effects on both the healthcare infrastructure and the underlying population are significant, with an appraised annual cost of 600 billion dollars [2,3]. While the causes and risk factors for CVD are complex and multifaceted, lipid disorders such as dyslipidemias (DLP) are clearly associated with its pathological onset and are thus a leading focus of interest for clinicians in primary and secondary prevention. However, DLP management is intricate, and the understanding of the underlying mechanisms is critical for the development of more appropriate innovative therapies.

The Mediterranean diet has garnered considerable attention in the past decades given its beneficial impacts on cardiometabolic and cardiovascular health [4,5]. Indeed, Mediterranean diet intake acts on both healthy individuals and subjects with cardiovascular risk factors, resulting in favorable clinical outcomes and more particularly in improvement of lipid disorders. This positive impact may be due to its high polyphenolic content, derived from vegetables, grapes and olive oil. For example, olive oil brings out cardioprotective effects due the presence of a myriad of polyphenolic constituents, including phenolic acids (e.g., caffeic, syringic acids), flavonoids (e.g., apigenin, luteolin), secoiridoids (e.g., oleuropein) and lignin (tyrosol, hydroxytyrosol) [6]. These results have warranted widespread recommendations for the Mediterranean diet with respect to CVD prevention and management [4].

The purpose of this critical review is first to provide a comprehensive summary and update on lipid disorders and PLPs features. In a second step, we will emphasize the major roles of PLPs on both primary and secondary prevention, and discuss the potential mechanisms contributing to their various actions. Third, we will examine the use of PLPs as therapeutic agents while identifying new perspectives for future research.

## 2. Dyslipidemia

### 2.1. Definition of Dyslipidemia and Related Biomarkers

DLP is described as abnormal levels of circulating lipids, presenting a high risk for CVD development [7]. Its etiology can be primary (genetic) or secondary [diet, drugs, chronic diseases and metabolic disorders, including obesity, metabolic syndrome (MetS) and type 2 diabetes (T2D)] [8]. To define DLP, clinicians usually rely on rapid fasting lipid profile, which encompasses triglycerides (TG), total cholesterol (TC), high-density lipoprotein-cholesterol (HDL-C), low-density lipoprotein-cholesterol (LDL-C) and non-HDL-C. Oftentimes, further evaluation of additional lipoprotein particles is necessary to get an accurate diagnosis, requiring more time and expertise. They include chylomicron (CM), CM remnants, very-low-density lipoprotein (VLDL), small and dense LDL and Lp(a) [9,10]. Apolipoproteins (Apo), the structural components of lipoprotein particles, are also very informative for diagnosis, and in particular Apos (A1 and B-100), the major moieties of HDL and LDL, respectively. DLP, detrimental to human health, is identified by excessive concentration of TG, TC, VLDL, LDL-C, non-HDL-C, Lp(a), CM and CM-remnants, along with decreased levels of HDL-C. Moreover, the inadequate association of lipids (TG, free cholesterol, cholesteryl ester and phospholipids) with Apos (A-I, A-II, A-IV, B-48, B-100, C-II, C-III, E) disrupt the normal composition of the blood lipoproteins, which creates a shift towards an atherogenic lipoprotein phenotype, in essence a hallmark of DLP, and ultimately contributes to atherosclerosis [11].

### 2.2. Chylomicron Formation and Postprandial Dyslipidemia

**Figure 1:**
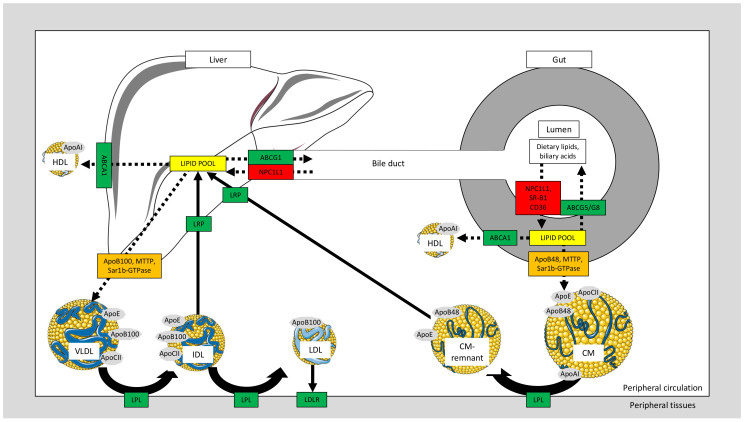
Lipid absorption, excretion and transport by the gut–liver axis. Intestinal lipids contained in diet and in biliary acids (originating from the liver and delivered into intestinal lumen through the bile duct) are absorbed by the small intestine following digestion and uptake by protein transporters: Niemann-Pick-C1-like-1 (NPC1L1), scavenger receptor B-1 (SR-B1) and cluster of differentiation-36 (CD36). In the enterocyte, lipids and apolipoproteins (Apo) are assembled into chylomicrons (CMs), a process requiring the essential proteins Apo B-48, microsomal triglyceride transport protein (MTTP) and Sar1b-GTPpase. Subsequently, CM are secreted into the peripheral circulation where their triglyceride (TG) components undergo lipolysis by lipoprotein lipase (LPL) after activation by Apo C-II. The resulting CM remnants are internalized by the liver following recognition by the specific low-density lipoprotein-like receptor protein (LRP). For their part, very-low-density lipoproteins (VLDLs) are assembled in the liver and released into the circulation to release fatty acids for peripheral tissues after hydrolysis by LPL.VLDL remnants or intermediate-density lipoprotein (IDL) can be taken up by liver receptors or be further metabolized into low-density lipoproteins (LDL) for cholesterol delivery in peripheral tissues through interaction with their LDL receptor (LDLR). On their side, high-density lipoproteins (HDL) are derived from the intestine and liver with the involvement of ATP-binding cassette transporter A1 (ABCA1) and Apo A-I. HDL confers atheroprotection via the process of reverse cholesterol transport whereby excess intracellular cholesterol is transported to the liver by HDL particles. Full arrows represent lipoprotein metabolism. Dotted arrows represent lipid movement. Boxed enzymes and receptors in green are associated with reduction of cholesterolemia, whereas red boxes are associated with increase in cholesterolemia. Some images in this figure were obtained and modified from Servier Medical Art (https://smart.servier.com).

One of the major functions of the small intestine is the transport of alimentary fat in the form of CM. Following the digestive phase involving bile acids and pancreatic lipase, the lipolytic products are absorbed by the enterocyte where they undergo lipid esterification along with the synthesis and post translational modification of different Apos, followed by the packaging of lipid and Apo components into lipoprotein particles [12,13,14,15,16]. Three key proteins should be given particular prominence: Apo B-48, microsomal triglyceride transport protein (MTTP) and Sar-1b (a GTPase protein) [17,18,19,20,21]. MTTP shuttles TG, cholesteryl ester and phospholipids to Apo B-48 in the endoplasmic reticulum, allowing packaging of CM particles, which are then exported to the Golgi for maturation under the control of Sar-1b. CMs are targeted to the basolateral site of the enterocyte in order to enter blood via the lymphatic duct. In the systemic circulation, lipoprotein lipase (LPL) hydrolyzes CM-TG in order to provide peripheral tissues with fatty acids (FA)s. Thereafter, CM remnants are mostly incorporated into the liver through Apo E recognition by hepatocyte receptors (Figure 1) [22].

Mounting evidence underlines the link between increased intestinal CM production in response to Western diet and atherosclerosis [23,24]. Indeed, raised levels of CM and their remnants correlate to intima media thickness and accelerated atherogenic process in MetS, insulin resistance (IR) and T2D conditions [25,26,27]. The chylomicronemia syndrome may also result from a deficiency in LPL, Apo CII or its associated proteins, leading to autosomal recessive disorder Type I hyperlipoproteinemia (HLP) [8,28]. Additional factors contributing to DLP from molecular aberrations of LPL and CM-remnant receptors or consequently to polymorphisms of LPL, Apo E, Apo B and MTTP [29,30,31,32,33,34].

### 2.3. Intestinal Cholesterol Transporters and Relation to Dyslipidemia

As dietary cholesterol (CHOL) intake contributes to plasma CHOL levels, which are associated with excessive CHOL deposition in the arterial intima, much attention has been paid to intestinal CHOL transporters [35]. The absorption of CHOL by enterocytes is controlled by (i) CHOL uptake at the apical site by Niemann-Pick-C1-like-1 (NPC1L1), scavenger receptor B-1 (SR-B1) and cluster of differentiation-36 (CD36) [36,37,38]; (ii) CHOL uptake at the basolateral site by the regulatory system composed of LDL receptor (LDLR) and proprotein convertase subtilisin/kexin type 9 (PCSK9); (iii) CHOL excretion at the apical site by the heterodimer of ATP-binding cassette transporters G5/G8; (iv) CHOL output at the basolateral site by ATP-binding cassette transporter A1 (ABCA1), which transfers cellular CHOL to lipid-poor Apo A-1 for nascent HDL formation; and (v) the trans-intestinal CHOL excretion pathway which significantly enhances neutral sterol excretion in humans (Figure 1). Noteworthy, important intra-enterocyte proteins, including acyl-CoA: cholesterol O-acyltransferase 2 (catalyzing the esterification of CHOL with FA) and hydroxylmethylglutaryl-CoA reductase (the rate-limiting enzyme in CHOL biosynthesis) are highly involved in intestinal CHOL absorption [35].

As reviewed above, the small intestine displays absorptive and excretory functions to modulate CHOL fluxes across the intestine, thereby favoring body CHOL homeostasis. Increased CHOL uptake (in response to upregulation of NPC1L1, SR-B1, CD36 and LDLR) or decreased CHOL excretion (due to downregulation of ABCA1, ATP-binding cassette transporter G5/G8 and trans-intestinal CHOL excretion) can influence pathogenesis of hypercholesterolemia, DLP and atherosclerosis [39,40,41,42,43]. Additionally, the transporters mediating intestinal CHOL absorption may act as determinants of Apo B-containing atherogenic lipoproteins [44,45].

### 2.4. VLDL Metabolism and Relation to Dyslipidemia

Similarly to CM assembly by the enterocyte, VLDL formation in the hepatocyte involves MTTP and Sar1b, while Apo B-100 becomes the major protein component [46]. After entering the bloodstream, VLDL-TG are hydrolyzed by LPL, thus releasing FA. VLDL-remnants, or more specifically intermediate-density lipoprotein, can either by recycled back into the liver or undergo further lipolysis to be converted into LDL particles for CHOL delivery to peripheral tissues via LDLR-Apo B-100 interaction (Figure 1). 

Defects or deletion of *LDLR* or *ApoB100* genes result in abnormally low uptake of LDL by various organs, particularly the liver. Consequently, there is a steep accumulation of circulating LDL-C, a condition known as familial hypercholesterolemia (FH), which is associated with aggravated risk of LDL deposition in the vessel wall and atherosclerosis occurrence. Indeed, FH is characterized by an autosomal dominant pattern of inheritance and can result in heterozygosity (2- to 3-fold increase in circulating LDL-C) or more serious homozygotic form (3- to 6-fold LDL-C elevation). Homozygous patients develop atherosclerosis and stenosis (e.g., coronary artery disease, calcifications in the aortic root and ascending aorta, aortic regurgitation, and even death) during the first two decades of life. 

Another causative gene in FH encodes *PCSK9*, which targets *LDLR* for degradation. Mutations or polymorphisms of *PCSK9* are a common cause of FH, where gain-of-function *PCSK9* mutations can cause familial autosomal dominant hypercholesterolemia. Moreover, rare mutations in *LDLR adapter protein 1*, *ApoE* p.Leu167del, or lysosomal acid lipase genes can mimic FH (Table 1) [8,28,47,48].

### 2.5. Additional Congenital Types of Primary Hyperlipoproteinemia

Apo E constitutes an important component of CM- and VLDL-remnants and is also a ligand for their receptor-mediated hepatic uptake. *Apo E* deficiency or genetic variants may lead to familial Type III HLP. *Apo E2* (substitution of cysteine in the normal *Apo E3 variant* for arginine at residue 158) interacts poorly with LDLR and LDLR-related protein, thereby DLP and an increased risk for atherosclerosis [28].

Type IV HLP is characterized by fasting hypertriglyceridemia due to a genetic autosomal dominant defect that increases VLDL secretion, raising the risk of abnormal glucose tolerance, athero-eruptive xanthoma, pancreatitis and CVD. Obesity, MetS, T2D, hypopituitarism, contraceptive steroids and glycogen storage disease I are secondary causes that can all trigger the development of Type IV HLP [8]. 

Lastly, type V HLP, also known as combined/mixed hypertriglyceridemia, is characterized by increased amounts of plasma CM and VLDL and decreased LDL and HDL after overnight fasting. In these conditions, TG levels are considerably elevated and enhance the risk of acute pancreatitis. Some patients exhibit high TC concentrations that can be accounted for increased VLDL [28]. Complete assessment of patients with Type V HLP also involves family sampling to discern the presence of familial Type V. In addition to primary Type V, secondary Type V has been noted and its development involves a multitude of metabolic derangements, including low TG clearance and/or their increased output aggravated by obesity, IR, T2D, alcohol intake, or the use of some hormones [8].

### 2.6. HDL Metabolism

#### 2.6.1. HDL Synthesis and Functions

**Figure 2:**
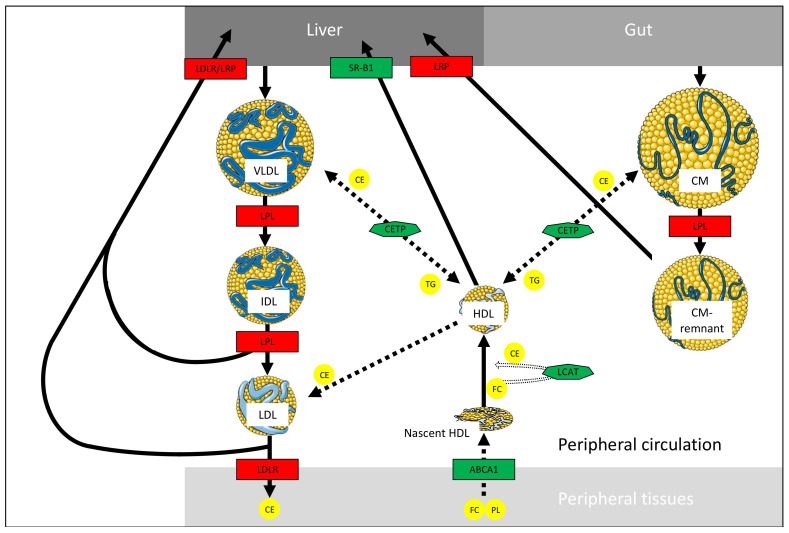
Interaction and lipid exchanges amidst circulating lipoproteins. As very low-density lipoprotein (VLDL) and chylomicron (CM) are secreted by liver and gut, respectively, lipolysis by lipoprotein lipase (LPL) releases fatty acids for peripheral tissues. VLDL- and CM-remnants are captured by the liver following recognition of their apolipoprotein (Apo) E content by low-density lipoprotein-like receptor protein (LRP). A small proportion of intermediate-density lipoprotein (IDL) particles can be directly taken by the liver (via LRP) while circulating IDL-triglyceride (TG)s are degraded by LPL to provide low-density lipoprotein (LDL) particles. The latter are the major carriers of free cholesterol (FC) and cholesteryl ester (CE), which are delivered to peripheral tissues after LDL uptake via LDL receptors (LDLR). Peripheral efflux of FC and phospholipids (PLs) mediated by ATP-binding cassette transporter (ABCA1) towards lipid poor high-density lipoprotein (HDL) represents the first step for reverse cholesterol transport. Esterification of FC in HDLs by lecithin-cholesterol acyltransferase (LCAT) promotes HDL maturation and size. On the other hand, CE can also be exchanged for TG via cholesteryl ester transfer protein (CETP) with TG-rich lipoproteins (e.g., VLDL and CM) or LDL. At this stage, HDLs transfer their CE content to the liver via scavenger receptor B-1 (SR-B1) involvement. The reverse cholesterol transport process ends by the hepatic conversion of cholesterol into bile acids, which represents the only route of cholesterol elimination from the human body. Full arrows indicate lipoprotein metabolism. Dotted arrows indicate lipid movement. Green-boxed enzymes or receptors are associated with HDL metabolism, whereas red-boxed enzymes or receptors are linked with remaining lipoproteins. Some images in this figure were obtained and modified from Servier Medical Art (https://smart.servier.com).

The liver and intestine constitute two important sites for the formation of new HDL particles (Figure 1). The crucial step for HDL biogenesis depends essentially on *ABCA1*. Genetic mutations or overexpression of *ABCA1* result in diminished or raised circulating levels of HDL-C, respectively [47,48,49]. In addition to the contribution of hepatic and intestinal nascent HDL biogenesis, lipolysis of TG-rich lipoproteins (CM and VLDL) by LPL is another important source of HDL production, with mutual exchange of lipids through the action of cholesteryl ester transfer protein (Figure 2) [50,51].

**Figure 3:**
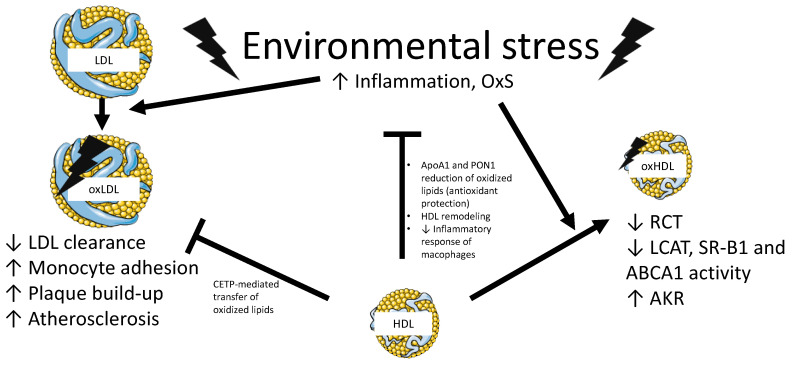
Properties of native and oxidized HDL with their impact on LDL particles. Excessive oxidative stress (OxS) and/or inflammation may transform circulating normal low-density lipoprotein (LDL) and high-density lipoprotein (HDL) particles into oxidized LDL (oxLDL) and oxidized HDL (oxHDL). Modified LDL and HDL may stay longer in the bloodstream given their impaired interaction with their specific receptors low-density lipoprotein receptor and scavenger receptor B-1 (SR-B1), respectively. Their diminished clearance contributes to the onset of atherosclerosis. Primary or secondary dyslipidemia leading to elevated levels of LDL exacerbate this problem, especially after transfer of oxidized lipids from oxLDL to oxHDL via cholesteryl-ester transfer protein (CETP). Beneficial apolipoprotein (Apo) A-1 or paraoxonase (PON)1 actions promote antioxidant and anti-inflammatory protection, which prevent lipid peroxidation magnification. Besides, accumulation of oxHDL is accompanied with aldo-keto reductase (AKR) activity alterations and loss of beneficial actions. ABCA1, ATP-binding cassette A1; LCAT, lecithin-cholesterol acyltransferase; RCT, reverse cholesterol transport. Some images in this figure were obtained and modified from Servier Medical Art (https://smart.servier.com).

The formation of CHOL-poor nascent HDL particles is achieved via the assembly of Apo A-1 and cellular lipids (mainly CHOL and phospholipids), a reaction catalyzed by ABCA1 [52,53]. The nascent discoidal HDL is progressively filled up in the blood circulation with CHOL transferred from peripheral tissues by ABCA1 and subsequently esterified to cholesteryl ester by lecithin cholesteryl ester transfer protein (LCAT) [54]. Filling with cholesteryl ester turns nascent HDL from a discoidal form into a spherical shape, and into larger HDL_3_ and much larger HDL_2_ particles. At this stage, HDL-C is removed by hepatic SR-B1, completing the reverse cholesterol transport process and ultimately ensuring that excess peripheral tissue CHOL is deposited in the liver for bile acids conversion [55,56,57]. The importance of HDL in protecting against CVD is attributed not only to its role in reverse cholesterol transport but also to its ability to act as an antioxidant, anti-inflammatory, anti-thrombotic, pro-vasodilatory and anti-apoptotic agent (Figure 3) [38,58,59,60,61].

#### 2.6.2. HDL-Related Disorders

Deficiency of HDL-C (hypoalphalipoproteinemia) can be a result of genetic defects of *ABCA1*, *LCAT* and *ApoA1*, which leads to Tangier disease, Fish-eye or Familial *LCAT* deficiency disease, and *ApoAI* deficiency or *ApoA1* variants [62]. Furthermore, genetic deficiency of cholesteryl ester transfer protein is characterized by markedly elevated levels of HDL-C while being associated with reduced atherosclerosis [63].

### 2.7. Treatment of Dyslipidemia

Depending on initial risk assessment, management of DLP primarily lies in prevention, and thus mostly consist of a therapeutic lifestyle approach where nutrition plays a key role [10]. However, if therapeutic goals cannot be reached through lifestyle, or in the event of a primary subtype of HLP, drug therapy or extracorporeal treatment is initiated (Table 2). Presently, the available medical arsenal for DLP mainly focuses on reducing LDL-C and non-HDL-C levels, but may act on Apo B, TG and HDL-C as well. Statins are the preferred choice for LDL-C lowering and are routinely prescribed as primary and secondary treatments [9,10]. If therapeutic goals still cannot be reached or if use of statins is contraindicated, secondary options typically include bile acid sequestrants, NPC1L1 inhibitors and PCSK9 antibodies [8,9,10,64,65,66,67,68,69].

As a first line of treatment, nutraceuticals with beneficial cardiometabolic properties such as PLPs are an interesting avenue in primary and secondary prevention of DLP, MetS, IR and atherosclerosis. General lack of undesired effects may make these phytochemicals particularly attractive and thus further warrants interest [70,71].

**Table 2:** Conventional treatments for dyslipidemia.

Treatment	Underlying Mechanism	Lipid Profile Variation (%)				Indication	CI Possible Adverse Effects	Reference
		TG	Non-HDL-C	LDL-C	HDL-C			
Pharmacological therapies								
Statins	HMG-CoA-R inhibitors	↓7–30	↓15–51	↓18–55	↑5–15	First line treatment	CI: Possible drug-drug interaction (3A4 inhibitors), pre-existent hepatic disease, end-stage kidney failure, heart failure (>class I on NYHA scale), pregnancy and/or breast-feeding. PAE: hepatic toxicity, myopathy, rhabdomyolysis, acute renal failure.	[9,10,64,65,66]
Bile acid sequestrants	Cholesterol chelation in gut’s lumen	↑0–10	↓4–16	↓15–30	↑3–5	Adjunct with statins or first line treatment if statins not recommended	PAE: GI symptoms, reduced effectiveness of other medications, increase in TG	[9,10,66]
Fibrates	PPARαagonist (VLDL secretion inhibition, LPL induction)	↓20–50	↓5–19	↓5–↑20	↑10–20	HyperTG	CI: Not recommended with statins	[9,10,66,67]
NPC1L1 inhibitors	Cholesterol absorption inhibitor	↓5–11	↓14–19	↓13–20	↑3–5	Adjunct with statins or first line treatment if statins not recommended	CI: Presence of an underlying hepatic disease	[9,10,66,67]
PCSK9 inhibitors	Inhibition of LDLR degradation			↓50		Adjunct with statins or first line treatment if statins intolerance	Injection site reactions	[10,66,71]
Mipomersen	Inhibition of Apo B synthesis			↓25		HyperTG related to acute pancreatitis	PAE: Hepatic steatosis	[64,65]
Lomitapide	Inhibition of MTTP in liver and gut			↓50		HyperTG related to acute pancreatitis	PAE: GI symptoms, hepatic steatosis	[64,65]
Non-pharmacological therapies								
Niacine	Increased expression/ activity of adipose LPL	↓20–50	↓8–23	↓5–25	↑15–35	HyperTG	CI: Not recommended with statins	[9,10,66,67]
Omega-3 fatty acids	PPARα agonist	↓19–44	↓5–14	↓6–↑25 ^1^	↓5–↑7	HyperTG	CI: Fish allergy	[9,66,67]
Dietary fibers	Delayed/ reduced cholesterol absorption			↓3–5		Primary prevention	PAE: GI symptoms	[72,73]
Monacolin	HMG-CoA-R inhibitor			↓0–20		Primary prevention	PAE: safety issues regarding presence of contaminants	[73]
Phytosterols	Cholesterol absorption inhibitor	↓4–9		↓7–10		Primary prevention		[66,74,75]

^1^ No LDL-C increase if omega-3 supplements contain exclusively eicosapentaenoic acid (no docosahexaenoic acid). CI, contraindication; DLP, dyslipidemia; GI, gastro-intestinal; HDL-C, high-density lipoprotein-cholesterol; HyperTG, hypertriglyceridemia; LDL, low-density lipoprotein; LDL-C, low-density lipoprotein-cholesterol; LDLR, low-density lipoprotein receptor; LPL, lipoprotein lipase; MTTP, microsomal triglyceride transport protein; NPC1L1, Niemann-Pick-C1-like-1; PAE, possible adverse effects; PCSK9, proprotein convertase subtilisin/kexin type 9; PPARα, peroxisome proliferator-activated receptor alpha; TG, triglycerides; VLDL, very low-density lipoprotein.3.

## 3. Polyphenols and Metabolic Benefits

### 3.1. Polyphenol Background

**Figure 4:**
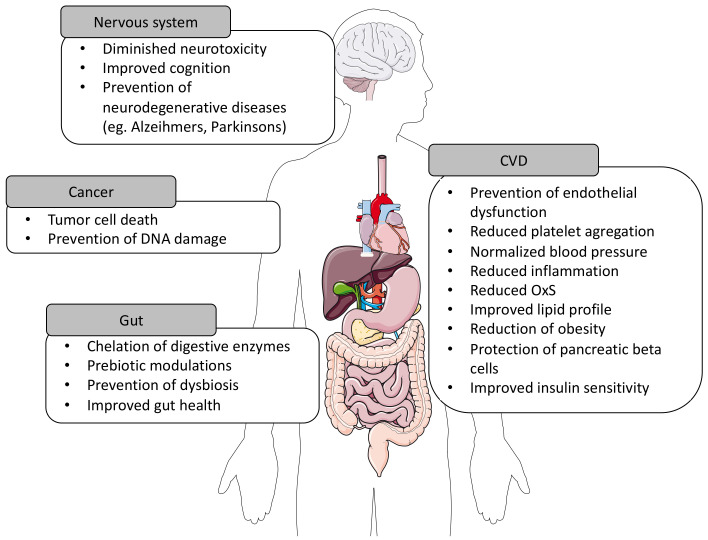
Potential health benefits of dietary polyphenols in chronic diseases. CVD, cardiovascular disease; OxS, oxidative stress. Some images in this figure were obtained and modified from Servier Medical Art (https://smart.servier.com).

**Figure 5:**
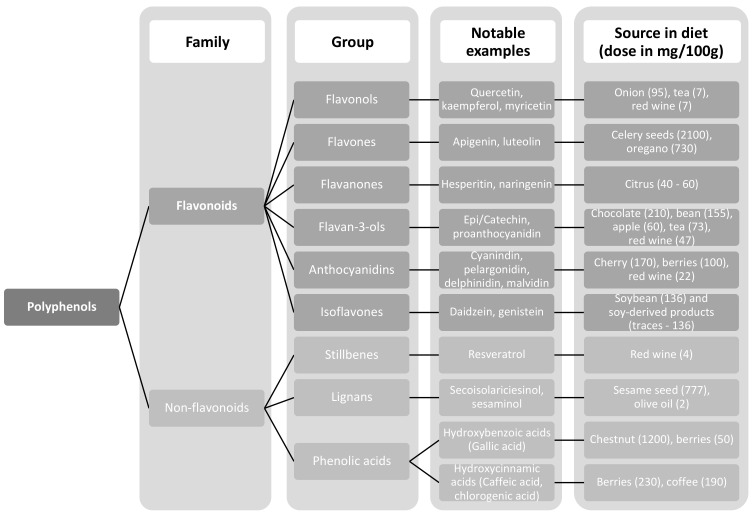
Classification of polyphenols.

PLPs represent a vast heterogenous class of phytochemicals. To date, more than 8000 compounds have been identified [70,76]. They are synthesized in plants as abundant secondary metabolites, which act as a powerful innate immunity agent, promoting both protection and survival [77]. Growing evidence underlines beneficial health properties of these natural polyphenolic compounds that work best for both prevention and therapy of multiple diseases (Figure 4). Their basic structure is represented by a benzene ring attached to one or more hydroxyl groups, thus forming the basic molecule upon which are grafted additional units, in association with organic acids and carbohydrates, to produce large polymers. PLPs are most commonly divided into two main families: flavonoids and non-flavonoids. The former is composed of diverse subgroups, including flavones, flavonols, flavan-3-ols, isoflavones, flavanones and anthocyanidins, whereas non-flavonoids regroup phenolic acids, lignans and stilbenes (Figure 5) [74,75,78].

### 3.2. Regulation of Oxidative Stress, a Component Affecting Metabolic Syndrome, by Polyphenols

Oxidative stress (OxS) arises when cellular antioxidant defense is exceeded by the overproduction of reactive oxygen species (ROS). The multitude of free radicals like superoxide anions, hydroxyl, peroxyl radicals, alkoxyl radicals and hydrogen peroxides threaten cellular integrity and homeostasis [79]. OxS persistence due to diverse mechanisms (e.g., mitochondrial respiratory chain, uncontrolled arachidonic acid cascade and NADPH oxidase, a membrane-bound enzymatic complex) adversely affects lipids, proteins and DNA nucleic acids, which ultimately leads to disruption of intracellular signaling, loss of physiological function, and cellular death [80,81]. As cell organelles are generally rich in iron, the Fenton’s reaction uses this transition metal to promote ample hydroxyl radical synthesis and biological damage [82]. Fortunately, inter- and intra-cellular antioxidant enzymes (e.g., superoxide dismutase, catalase, glutathione peroxidase and glutathione reductase) scavenge ROS and succeed in preventing OxS damage [80]. 

**Figure 6:**
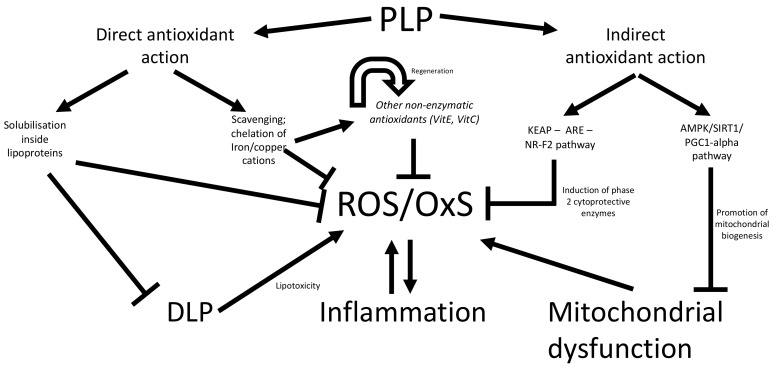
Antioxidant protection and metabolic actions of polyphenol supplementation. Polyphenols (PLPs) may protect against oxidative stress (OxS) through either direct or indirect antioxidant mechanisms. In the former, PLPs can scavenge and neutralize ROS before the occurrence of oxidative damages to lipids, proteins and DNA. Moreover, PLPs have also the capacity to promote protection by regenerating additional exogenous antioxidants such as tocopherol and ascorbic acid. Solubilization and transport of PLPs by lipoproteins such as chylomicron or high-density lipoprotein/low-density lipoprotein particles may specifically prevent OxS derived from dyslipidemia (DLP) and cardiometabolic complications, notably atherosclerosis. Indirectly, PLPs stimulate endogenous antioxidants, including the regulatory KEAP-ARE-NRF2 pathway, in order to enhance the synthesis of phase 2 cytoprotective enzymes (e.g., γ-glutamylcysteine synthetase, glutathione peroxidase, glutathione reductase, glutathione S-transferase, NAD(P)H:quinone oxidoreductase 1, thioredoxin, thioredoxin reductase, catalase and superoxide dismutase). Furthermore, PLPs can stimulate the AMPK/SIRT1/PGC-1α pathway for prevention of mitochondrial dysfunction. AMPK, AMP-activated protein kinase; ARE, Antioxidant response element, KEAP, Kelch-like ECH-associated protein; NR-F2, nuclear factor erythroid-derived 2-like 2; PGC1-α, peroxisome proliferator-activated receptor gamma coactivator 1-alpha; ROS, reactive oxygen species; SIRT1, sirtuin 1.

PLPs, owing to their chemical structure and ROS scavenging properties, have commonly been identified as direct antioxidants. Their properties have been extensively documented over the years, mainly through numerous in vitro studies [83,84,85]. Nevertheless, a common criticism is that the experimental model too heavily relies on elevated concentrations of unaltered PLPs, which may not accurately reflect in vivo conditions. Indeed, low bioavailability, chemical modification during digestion in the gut lumen (i.e., deglycosylation), post-absorption in the liver (methyl, glucuronide and sulfate conjugation), dubious bioaccessibility, and short biological half-life render such experimental high dosage unrealistic, and even create a rift between in vitro and in vivo experimental conditions by a factor of 10^3^–10^6^ [75]. Besides, in vitro models cannot account for the complex interaction of PLPs with the gut microbiota and resulting metabolites [75,76,83,84,86,87,88]. However, specific PLPs have garnered interest owing to their high bioavailability and consequently relatively high plasmatic concentrations. Prime examples, such as resveratrol or combined flavonoids and stilbene from red grapes, have shown clinical evidence of direct amelioration of redox imbalance by either restoring α-tocopherol levels or otherwise preventing its degradation by initial ROS first encounter (Figure 6) [85,89].

Several groups have documented the antioxidant ability of PLPs against OxS-induced damages either in chronic non-communicable diseases such as obesity, MetS, non-alcoholic fatty liver disease, T2D and CVD. A close association has been noted between obesity (the grimmest and most predominant public health threat worldwide) and OxS, which is characterized by NADPH oxidase upregulation and antioxidant enzyme downregulation [90]. High FA and glucose concentrations could account for these abnormalities, which are likely exacerbated by mitochondrial ROS formation [90,91,92]. PLPs were shown to fight OxS-antioxidant imbalance and avoid free radical injury, operating through direct or indirect antioxidant mechanisms of action (Figure 6). For example, supplementation of epigallocatechin gallate molecules, derived from green tea, not only served as singlet oxygen quenchers and enhanced endogenous antioxidant defenses but also mitigated hepatic lipid peroxidation and protein nitration while attenuating obesity-triggered steatosis through lowering hepatic and adipose tissue lipogenesis [93,94]. Moreover, it appeared in high-fat-induced obese rats that the advantageous impact of green tea PLP on body weight was produced via regulation of obesity-related anorectic genes, and anti-inflammation and antioxidant capacity [95]. For their part, nonflavonoid PLPs (e.g., resveratrol) inhibited oxygen-free radical formation by impeding NADPH oxidase-associated ROS production [96,97], a very important finding since elevated OxS is chiefly implicated in the pathogenesis of hypertension, DLP and CVD [98,99]. Resveratrol-mediated antioxidant upregulation and OxS lessening stimulated endothelial NO, while preventing inflammation and platelet aggregation [100,101]. Noteworthy, kelch-like ECH associated protein 1 and nuclear factor erythroid 2-related factor 2 system was the central cyto-protective mechanism for the decline of OxS elicited by PLPs (Figure 6) [102]. 

While long-lasting production of ROS induces strong IR, PLP antioxidants reduce ROS levels and exert glucose-lowering effects through amelioration of insulin sensitivity in humans and animal models of T2D [103,104,105,106,107]. In fact, OxS neutralization in various systems such as the muscle, adipose tissue and liver by PLPs contribute to the improvement of metabolic abnormalities [104]. Polyphenolic antioxidants may also serve to temper high blood pressure. For example, resveratrol prevented the development of hypertension and significantly lowered blood pressure in spontaneously hypertensive rats, possibly through the inhibition of Giα overexpression and other upstream signaling molecules [108,109].

DLP occurs very often in metabolic disorders and is associated with elevated OxS [110,111,112,113]. Available evidence has shown the susceptibility of lipids and lipoproteins, LDL in particular, to generate ROS [114,115]. Various studies documented high circulating levels of oxidized-LDL in MetS and T2D, as well as in FH [116,117]. Notably, PLPs such as curcumin exhibit hypolipidemic actions via various mechanisms while decreasing lipid peroxidation in lipoproteins. Specifically, PLPs have the potential to reduce circulatory CHOL and lipid peroxides, while increasing HDL-C in both animal models and clinical trial studies [118,119].

### 3.3. Regulation of Inflammation in Cardiometabolic Disorders by Polyphenols

As is the case for OxS, inflammatory processes are highly necessary for immunosurveillance and host defense since they respond to infectious agents, clear out necrotic cells and debris, and heal injuries and tissue damage [120]. However, if harmful triggers are persistent, inflammation can turn against healthy tissues, which become damaged and are even destroyed, thereby resulting in metabolic disorders and atherosclerosis [121]. Sustained low-grade inflammation predisposes to MetS components in view of raised levels of inflammatory cytokines, along with reduced concentrations of anti-inflammatory adiponectin [122]. This chronic inflammatory state could affect vascular and endothelial functions via nitric oxide lowering and ROS elevation [123]. Noteworthy, interactive relationship between OxS and inflammation constitutes the driving force for the severity of cardiometabolic complications and CVD [124]. Their interplay activates immune cell responses; recruits leukocytes, chemokines and adhesion molecules; and triggers vascular dysfunction via the activation of NADPH oxidase, xanthine oxidase and mitochondrial ROS overproduction. Collectively, these events contribute to both vascular and functional abnormalities, notably atherosclerosis progression and thrombus formation [125,126]. Furthermore, the simultaneous rise in inflammatory and oxidative status induces pathogenic mechanisms, favoring atherogenic lipoprotein production, oxLDL formation, atherogenic Apo B-containing lipoprotein retention in sub-endothelium, and HDL dysfunction [127,128,129]. 

On the other hand, down-regulation of pro-inflammatory cell signaling modulators, such as the nuclear factor-κB, the activated protein-1 and the mitogen activated protein kinases along with upregulation of nuclear factor-κB cytoplasmic inhibitor IκB, have been proposed as potential mechanisms of action of PLPs [130]. Further, PLPs may operate through inhibition of the arachidonic cascade and derivative eicosanoids [130,131]. Eicosanoids serve a modulating drive to the physiological inflammatory response, and consequently several anti-inflammatory drugs operate by limiting and/or inhibiting their production [132]. PLPs may block cellular release of arachidonic acid and/or inhibit the enzymes implicated in the cyclooxygenase pathway [130,131]. 

### 3.4. Polyphenols Counteract Cardiometabolic Complications by Regulating the Gut Microbiota

The gut microbiota has emerged in recent years as a novel and key player for metabolic diseases. Indeed, dysbiosis is associated with impaired gut integrity, local and systemic inflammation, OxS, reduced satiety, increased adiposity and ectopic lipid deposition [133]. On the other hand, healthy diets can favorably alter the composition of intestinal bacteria, which in turn promotes energy balance and body weight control, thereby reducing the risk of developing cardiometabolic complications [134]. In such cases, bacteria increase the production of short-chain FAs, such as acetate, propionate and butyrate, acting as signaling molecules and resulting in “energy harvest”. For example, butyrate provides a desirable energy source for colonocytes in the gut, improves mucus function via increased mucin production, strengthens gut barrier defense integrity via the promotion of tight junction proteins and stimulates gastrointestinal peptide secretion for improved insulin secretion and satiety [135,136,137]. Further, commensal bacterial colonization in the gut could either limit the invasion of nefarious species or halt their spread, therefore limiting the local production of pathogenic metabolites such as LPS and gut immunity overstimulation leading to metabolic endotoxemia and inflammation [138,139].

**Figure 7:**
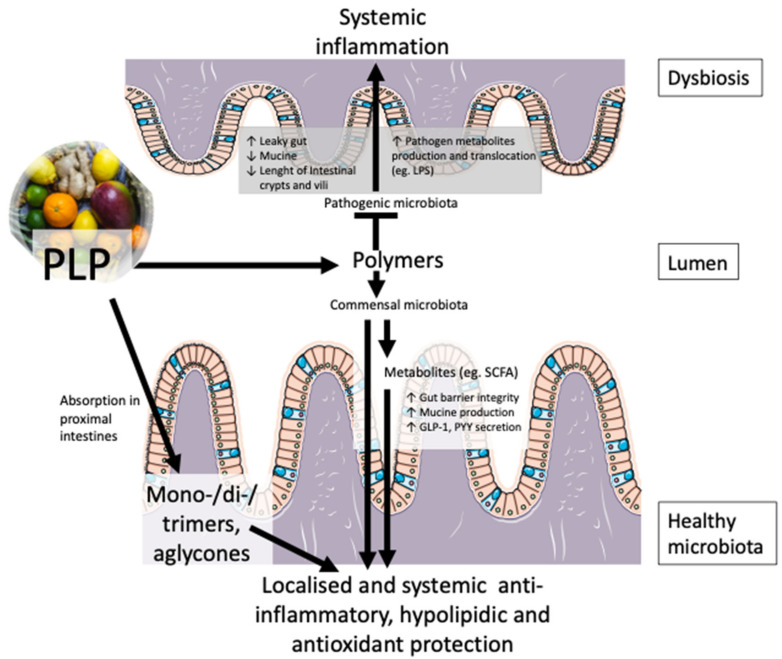
Protection against inflammation and microbiota dysbiosis by polyphenols. Polyphenols (PLP) included in diet may be composed of a wide range of molecules, from monomers to polymers. While the smaller mono-/di- and trimers can be directly absorbed in the proximal intestine, polymers need to continue their transit to undergo catabolism by microbiota in the colon. Commensal bacteria and their metabolites promote a wide variety of beneficial metabolic outcomes for local epithelial cells. Advantageous actions include improvement of gut integrity and production of both mucus and of gastrointestinal peptides. In addition, control over microbiota dysbiosis adverts the formation of nefarious metabolites such as bacterial lipopolysaccharide (LPS), thereby enhancing anti-inflammatory protection, and limiting further environmental stressors such as oxidative stress and lipid metabolism dysregulation. GLP-1, glucagon-like peptide 1; PYY, peptide YY; SCFA, short-chain fatty acids. Some images in this figure were obtained and modified from Servier Medical Art (https://smart.servier.com).

The polymeric fraction of PLP, indigestible and unabsorbable in the proximal intestine, interacts with colonic microflora, thereby increasing production of beneficial metabolites such as short-chain FAs and stimulating their advantageous effects (Figure 7). Animal models challenged with PLPs exhibit a high production of beneficial bacterial population such as *Akkermansia municiphilia* Sp. with a significant amelioration of inflammatory markers, gut permeability and insulin sensitivity [135,140,141,142]. Further, safeguard of a metabolically healthy gut–liver axis via microbiota reconfiguration may present an interesting avenue in the lipid-lowering potential of PLPs.

## 4. Methods

The available literature regarding polyphenol challenge on lipid profile in humans has been searched and analyzed. Electronic database Medline (PUBMED) was used for article research. The following keywords were employed: “polyphenols”, “lipids”, “triglycerides”, “cholesterol”, “LDL-C” and /or “HDL-C”. In order to be considered eligible, clinical trials had to comprise at least one parameter of this lipid profile, challenged by PLP intake and with the indication of lipid levels at baseline and following intervention. No discrimination was made regarding publication date, number or sex of participants enrolled. We excluded trials focusing on other pathologies. We also rejected meta-analyses, reviews and animal studies. Thus, 49 published clinical trials corresponding to our selection criteria were retained and thoroughly analyzed.

## 5. Polyphenol Supplementation in Humans—Intervention Trials

Recently, interest in dietary PLPs for human health has led to a great deal of research, especially in the cardiometabolic field. The following sections of this review particularly cover in vivo clinical studies, with a special focus on the potential of PLP to treat various types of human DLP.

### 5.1. Chronic Intake Interventions

Trials lasting more than a single time point (2 weeks to a whole year) were considered as a chronic intake/supplementation of PLP. The clinical status of the anthropometric and biochemical markers of participants enrolled, in essence their cardiometabolic state and overall level of risk, may severely impact their responsiveness to treatment and therefore account for the extensive amount of variability in clinical trials [136]. Further, since DLP is a pathology not only associated with the MetS, but which serves as a defining risk factor for diagnosis as well, the qualitative and quantitative assessment of other risk factors at play (namely abdominal obesity, systolic high blood pressure and IR) could thereby better reflect the initial metabolic state of participants enrolled. This could in turn potentially provide a better indication for the preventive use of PLP supplementation. 

#### 5.1.1. Impact of Polyphenols on Healthy Participants

The hypothesis of the first set of studies was that the chronic consumption of PLP-rich nutrients would maintain or improve the lipid profile of healthy participants while ensuring safety and lack of side effects. In total, 15 clinical trials are summarized in Table 3. Subjects were between the ages of 26 and 42, with a mean age of 34. The most relevant lipid findings in response to PLP indicate a decrease in TG in 8/12, TC in 7/12 and LDL-C in 8/11 along with an increase in HDL-C in 6/10 studies. The other clinical trials showed opposite trends. Although side effects have not been reported by the different groups, the results do not provide a clear picture of the favorable effect of PLPs on lipid and lipoprotein parameters in healthy individuals. The inconsistency may stem from the study design, huge PLPs concentration (45.3–3589 mg/day), intake duration (14–168 days), and matrix (Table 3). Importantly, food matrix represents one of the major limiting factor affecting PLP bioavailability and subsequent gastrointestinal absorption [84]. Further studies are certainly warranted to investigate the preventive actions of PLP in healthy individuals. Above all, efforts are needed in order to distinguish the specific effects of PLPs versus those of the many pro-health components (e.g., vitamins, fibers, functional food products, and minerals) present in fruits, vegetables and plants. This is a central aspect for understanding the ultimate contribution of PLPs, which will provide a cost-effective and safe alternative for the prevention of lipid disorders.

#### 5.1.2. Impact of Polyphenols on Dyslipidemia

As there presently lacks any studies examining PLP challenge on any primary subtypes of DLP, the following sections address clinical interventions including participants presenting any subtype of secondary DLP. To this end, it was important to consider the baseline lipid profile, which had one of the following criteria: hypertriglyceridemia (>1.7 mM), hypercholesterolemia (>5.2 mM), elevated LDL-C (>3.4 mM) and/or low levels of HDL-C (<1 mM for men/1.3 mM for women) [10,150]. We then analyzed whether study participants with high CVD risk were more likely to benefit from PLP challenge. Importantly, studies were further separated based on the number of co-morbidities accompanying DLP, which ranges from one to three (Table 4, Table 5 and Table 6), and by including the CVD risk based on the Framingham risk score. The latter was chosen since it takes into account the age, the levels of TC and HDL-C, systolic blood pressure and presence of T2D. 

##### Impact of Polyphenols on Patients with a Single Dyslipidemia Component

In the 15 of clinical trial studies listed with a single component of DLP (Table 4), an improvement is noted in TG (*n* = 10), TC (*n* = 8) and HDL-C (*n* = 11) in response to PLP treatment in the majority of clinical trials. However, mixed results were observed in LDL-C. The variability of the findings may be due to the divergences in the study design involving PLP type, the matrix (as method of delivery) and dosage (0.05–2148 mg/d), as well as trial length (15–56 days), number of patients (20–184) and gender (number of women limited to 0–21).

##### Impact of Polyphenols on Patients with Two Dyslipidemia Components

Intriguingly, only 8 studies are available in this DLP category involving two morbidities (Table 5). Despite the limited number, it is possible to observe an improvement in TG (6/8), TC (5/8), LDL-C (5/8) and HDL-C (6/8). We can observe a great variability in the dose of PLPs (40–1500 mg/day), number of patients (*n* = 8–43), age of subjects (42–62 years), duration of the trial (14–77 days), matrix and gender.

##### Impact of Polyphenols on Patients with Three Dyslipidemia Components

The majority of trials in this category of patients showed an improvement of TG (8/10), TC (8/10, LDL-C (8/10) and HDL-C (6/9) in response to PLPs (Table 6). Noteworthy, in this category of DLP with three morbidity factors, two studies using resveratrol yielded poor results. Findings of a few studies were controversial as revealed in a meta-analysis conducted by Zhang et al. who found that resveratrol supplementation significantly increased total- and LDL-C concentrations [151]. Nevertheless, we expected hypocholesterolemic effects of resveratrol given positive findings in mice, including CHOL lowering and atherosclerosis protection via enhanced activity of peroxisome proliferator-activated receptor α [152], improvement of the endothelial activity [153], suppression of platelet aggregation [154], and reduction of blood pressure [155].

**Table 4:** Clinical studies evaluating lipid/lipoprotein status of dyslipidemia participants with a single morbidity in response to polyphenol supplementation.

Polyphenols			Protocol		Participants										Variation of Lipid Profile ^1^				Reference
Dietary Source (Main PLPs) ^2^	Dosage (mg/Day)	Matrix	Intake Repartition	Length ^S.D.^ (Days)	*n* (Female)	Age ^3^ (Years)	D.-O. (%)	↑LDL- C	↑TG	↓HDL- C	Obesity	IR	↑SBP	FRS (%)	TG	TC	LDL- C	HDL- C	
Coffee (hydroxycinnamic acids, methylxanthines)	510.6	Diet (drink)	Tid	56 ^CO^	27 (17)	33.7 ± 1.8	4	√						0.4	↓20% *	N/A	N/A	↓1%	[149]
Virgin olive oil (lignans)	2.9	Diet	Die	21 ^CO^	33 (14)	55.2 ± 1.8	15	√						9.5	↓6%	↓5%	↑1%	NV	[156]
Enriched virgin olive oil (hydroxytyrosol derivatives, lignans, flavonoids)	12.1	Diet	Die	21 ^CO^	33 (14)	55.2 ± 1.8	15	√						9.5	↑3%	↓4%	NV	↑2%	[156]
Enriched virgin olive oil (hydroxytyrosol derivatives, lignans)	12.6	Diet	Die	21 ^CO^	33 (14)	55.2 ± 1.8	15	√						9.5	↑3%	↓4%	↓8% *	↓1%	[156]
Olive oil (not specified)	0.05	Diet	Die	21 ^CO^	182 (0)	33.3 ± 0.8	8			√				1.9	↓6%	NV	↑1%	↑2% *	[157]
Olive Oil (not specified)	3.6	Diet	Die	21 ^CO^	184 (0)	33.3 ± 0.8	8			√				1.9	↓4%	NV	↑1%	↑3% *	[157]
Olive Oil (not specified)	8.1	Diet	die	21 ^CO^	183 (0)	33.3 ± 0.8	8			√				1.9	↓5%	NV	↑2%	↑4% *	[157]
Pine Bark (flavonoids)	150	Capsule	Die	42 ^CO^	25 (15)	30.0 ± 1.5	0			√				1.7	↑2%	↓2%	↓7% *	↑11% *	[158]
Cocoa (epicatechin, catechin, procyanidin)	282	Diet (drink)	Bid	28 ^P^	37 (21)	49.9 ± 1.3	0	√						5.5	↓7%	↓3%	↓5% *	↑9% *	[159]
Cocoa (epicatechin, catechin, procyanidin)	211	Diet (drink)	Bid	28 ^P^	32 (18)	49.9 ± 1.3	0	√						5.4	↓2%	↓2%	↓4% *	↑7% *	[159]
Cocoa (epicatechin, catechin, procyanidin)	141	Diet (drink)	Bid	28 ^P^	31 (18)	49.9 ± 1.3	0	√						5.5	NV	↓3% *	↓5% *	↑5% *	[159]
Chocolate (flavanol, epicatechin) + fibers	45.3	Diet (drink)	Bid	28 ^CO^	20 (11)	30.0 ± 6.2	12	√						2.0	↑1%	↑2%	NV	↑12% *	[146]
Dark chocolate (not specified)	2148	Diet	Die	15 ^CO^	92 (34)	45.0 ± 1.1	40	√						5.9	↓8%	↑2%	↑4%	↑5% *	[160]
Dealcoholized red wine (not specified)	1000	Diet (drink)	Die	42 ^P^	15 (15)	57.6 ± 1.3	0	√						6.3	↓2%	↓1%	NV	↓5%	[86]
Mate Tea (chlorogenic acid)	107	Diet (drink)	Die	15 ^CO^	92 (34)	45.0 ± 1.1	40			√				5.9	↓3%	↑1%	↑3%	↑1% *	[160]

^1^ Change as percentage of baseline. Up and down arrows indicate lipid/lipoprotein increase and decrease, respectively, following PLP challenge. * indicates significant variation (*p* < 0.05). ^2^ As specified by the authors in the case of a non-purified extracts. ^3^ Values represent mean ± standard error of the mean. Bid, twice a day; ^CO^, cross-over intervention; Die, daily; D.-O., drop-out rate; FRS, Framingham risk score; HDL-C, high-density lipoprotein cholesterol; IR, insulin resistance; LDL-C, low-density lipoprotein cholesterol; N/A, not available; NV, no variation; ^P^, parallel intervention; PLP, polyphenol; SBP, systolic blood pressure; S.D., Study design; TC, total cholesterol; TG, triglycerides; Tid, thrice a day.

**Table 5:** Clinical studies evaluating lipid/lipoprotein status of dyslipidemia participants with two morbidities in response to polyphenol supplementation.

Polyphenols			Protocol		Participants										Variation of Lipid Profile ^1^				Reference
Dietary Source (Main PLPs) ^2^	Dosage (mg/Day)	Matrix	Intake Repartition	Length ^S.D.^ (Days)	*n* (Female)	Age ^3^ (Years)	D.-O. (%)	↑LDL- C	↑TG	↓HDL-C	Obesity	IR	↑SBP	FRS (%)	TG	TC	LDL- C	HDL-C	
Carob (not specified) +7.2 g insoluble fibers	40	Capsule	Bid	30 ^P^	43 (22)	42.9 ± 9.5	9	√		√				6.6	↓23% *	↓18% *	↓23% *	↑6% *	[161]
Red grape (anthocyanidins, quercetin, myricetin)	640	Juice	Bid	14 ^P^	26 (13)	62.0 ± 3.4	10	√		√				12.8	↓8%	↓11% *	↓18% *	↑13% *	[89]
Red wine (not specified)	1000	Diet (drink)	Die	42 ^P^	14 (14)	58.4 ± 1.3	0	√					√	7.3	↑17%	NV	↓8% *	↑17% *	[86]
Catechins, theaflavins	224.4	Capsule	Die	77 ^P^	31 (11)	50.1 ± 0.5	0	√	√					7.0	↓13%	↓1% *	↓2% *	↑3%	[162]
Theaflavins	77.5	Capsule	Die	77 ^P^	34 (12)	47.5 ± 1.0	0	√	√					7.0	↑7%	↓3% *	↓7% *	↑2%	[162]
Resveratrol	1500	Capsule	Bid	14 ^CO^	8 (0)	45.8 ± 3.1	0		√		√			6.7	↓20%	N/A	N/A	N/A	[163]
Resveratrol	150	Capsule	Die	30 ^CO^	18 (11)	50.4 ± 2.0	18	√			√			5.4	↓8%	↓4%	↑1%	↑3%	[85]
Cranberry (proanthocyanidins, anthocyanidins)	346	Diet (drink)	Bid	56 ^P^	29 (15)	76.6 ± 1.6	12		√	√				4.7	↓8%	NV	↑1%	↓3%	[164]

^1^ Change as percentage of baseline. Up and down arrows indicate lipid/lipoprotein increase and decrease, respectively, following PLP challenge. * indicates significant variation (*p* < 0.05). ^2^ As specified by the authors in the case of a non-purified extracts. ^3^ Values represent mean ± standard error of the mean. Bid, twice a day; ^CO^, cross-over intervention; Die, daily; D.-O., drop-out rate; FRS, Framingham risk score; HDL-C, high-density lipoprotein cholesterol; IR, insulin resistance; LDL-C, low-density lipoprotein cholesterol; N/A, not available; NV, no variation; ^P^, parallel intervention; PLP, polyphenol; SBP, systolic blood pressure; S.D., Study design; TC, total cholesterol; TG, triglycerides.

**Table 6:** Clinical studies evaluating lipid/lipoprotein status of dyslipidemia participants with three morbidities in response to polyphenol supplementation.

Polyphenols			Protocol		Participants										Variation of Lipid Profile ^1^				Reference
Dietary Source (Main PLPs) ^2^	Dosage (mg/Day)	Matrix	Intake Repartition	Length ^S.D.^ (Days)	*n* (Female)	Age ^3^ (Years)	D.-O. (%)	↑LDL-C	↑TG	↓HDL-C	Obesity	IR	↑SBP	FRS (%)	TG	TC	LDL-C	HDL- C	
Bergamot PLP (neoeriocitrin, naringin, neohesperidin) (+ statin)	1000	Capsule	Die	30 ^P^	15 (N/A)	N/A	0	√	√	√				>20	↓36% *	↓38% *	↓53% *	↑37% *	[165]
Bergamot PLP (neoeriocitrin, naringin, neohesperidin)	1000	Capsule	Die	30 ^P^	15 (N/A)	N/A	0	√	√	√				>20	↓31% *	↓31% *	↓41% *	↑18% *	[165]
Amla (Indian gooseberry) (not specified)	350	Capsule	Bid	84 ^P^	49 (27)	40.7 ± 1.6	0	√	√	√				5.5	↓34% *	↓24% *	↓20% *	↓10% *	[166]
Chokeberry (anthocyanidins)	772	Diet (drink)	Die	28 ^P^	23 (11)	47.5 ± 1.5	0	√	√				√	6.7	↓19% *	↓4%	↓7%	↓1%	[167]
Yerba mate tea (green or roasted) (cholorogenic acid, 4,5-dicaffeolquinic acid, gallocatechin)	3589	Diet (drink)	Tid	20 ^P^	57 (34)	45.8 ± 1.6	12	√	√	√				3.9	↓3%	↓3%	↓8% *	↑4% *	[144]
Yerba mate tea (green or roasted) (cholorogenic acid, 4,5-dicaffeolquinic acid, gallocatechin)	3589	Diet (drink)	Tid	40 ^P^	57 (34)	45.8 ± 1.6	12	√	√	√				3.9	↓3%	↓5% *	↓9% *	↑3%	[144]
Whole red grape (not specified) + 2.7 g of fibers	63	Diet	5x/day	56 ^P^	22 (18)	50.5 ± 1.5	0	√	√	√				9.5	↓1%	↓9% *	↓15% *	↓6%	[168]
Whole white grape (not specified) + 5.3 g of fibers	58	Diet	5x/day	56 ^P^	24 (18)	50.6 ± 1.3	0	√	√	√				7.1	↓4%	↓8% *	↓10% *	↓7%	[168]
Resveratrol	500	Capsule	Bid	30 ^P^	24 (12)	58.5 ± 3.4	0	√	√				√	12.8	↑20%	↑5% *	↑5%	↓2%	[169]
Resveratrol	3000	Capsule	Bid	56 ^P^	10 (0)	48.8 ± 1.7	0	√	√		√			18.4	↑31%	↑2%	↑9%	NV	[170]

^1^ Change as percentage of baseline. Up and down arrows indicate lipid/lipoprotein increase and decrease, respectively, following PLP challenge. * indicates significant variation. ^2^ As specified by the authors in the case of a non-purified extracts. ^3^ Values represent mean ± standard error of the mean. Bid, twice a day; ^CO^, cross-over intervention; Die, daily; D.-O., drop-out rate; FRS, Framingham risk score; HDL-C, high-density lipoprotein cholesterol; IR, insulin resistance; LDL-C, low-density lipoprotein cholesterol; N/A, not available; NV, no variation; ^P^, parallel intervention; PLP, polyphenol; SBP, systolic blood pressure; S.D., Study design; TC, total cholesterol; TG, triglycerides; Tid, thrice a day.

#### 5.1.3. Impact of Polyphenols on Metabolic Syndrome and Type 2 Diabetes

As the MetS is viewed as one of the most challenging health problems of our century, numerous scientists and clinicians are actively seeking effective drugs to reduce severity-associated co-morbidities, including T2D and CVD. Currently, the treatment of the MetS is based on multiple pharmacological agents directed against each of its components. Although lifestyle modification remains an interesting approach, many groups attempt to uncover novel effective nutraceuticals to alleviate its severity and development of cardiometabolic complications. Dietary PLPs have often been proposed as a powerful tool to fight the pathophysiological complexity related to both T2D and CVD. The goal in this following section is to determine whether PLPs may tackle T2D and CVD according to available clinical evidence.

Surprisingly, only 14 clinical trials have been conducted on the modulation of the lipid profile associated-MetS patients as a function of dietary PLPs. As noted in Table 7. About two thirds of these trials showed an improvement in TG, TC and LDL-C in response to PLPs supplementation, whereas a negative effect was observed on HDL-C. The large variability of the data could not recapitulate the results obtained in in vitro and preclinical investigations [171,172,173,174,175,176]. The discrepancy is likely due to the variation in the dose of PLPs (150–3000 mg/d), length of the studies (21–56 days), number of participants (10–68), female-male ratios (0–1) and food matrix. Therefore, future research is needed before supporting a potential role of PLPs in reducing lipid concentrations. The same holds true for HDL-C in view of its PLP-promoting increase reported by the work of various groups. Ultimately, PLP effectiveness demonstration in ameliorating DLP would be helpful towards integrating them in MetS treatment.

Although the number of clinical trials (*n* = 7) remains limited in investigating diabetic DLP in response to supplementation of PLPs, findings are more meaningful in T2D (Table 8) compared to MetS (Table 7). The majority of studies described their reducing effect on TG (13–42%), TC (2–12%) and LDL-C (1–15%), as well as their increasing effect on HDL-C (1–11%). Only resveratrol failed to alleviate diabetic DLP [177]. Diversity in length of supplementation (56–183 days) and delivery through capsules could account for the differences noted in PLPs effects. Long-term studies involving a large cohort of subjects together with careful diet control are needed in order to confirm the potential effect of PLPs on MetS and T2D in humans, without, of course, leaving aside genetic differences between populations.

**Table 7:** Clinical studies evaluating lipid/lipoprotein status of participants with metabolic syndrome ^1^ in response to polyphenol supplementation.

Polyphenols			Protocol		Participants										Variation of Lipid Profile ^2^				Reference
Dietary Source (Main PLPs) ^3^	Dosage (mg/Day)	Matrix	Intake Repartition	Length ^S.D.^ (Days)	*n* (Female)	Age ^4^ (Years)	D.-O. (%)	↑LDL-C	↑TG	↓HDL-C	Obesity	IR	↑SBP	FRS (%)	TG	TC	LDL- C	HDL- C	
Eckonia cava (not specified)	144	Diet (drink)	Bid	84 ^P^	32 (21)	40.2 ± 10.1	0	√		√	√	√		3.8	↓8%	↓9% *	↓14% *	↑13% *	[178]
Eckonia cava (not specified)	72	Diet (drink)	Bid	84 ^P^	33 (22)	40.6 ± 9.3	0	√		√	√	√		3.8	↓3%	↓7% *	↓10% *	↑9%	[178]
Grape (flavanols, anthocyanidins)	195	Diet (drink)	Bid	28 ^CO^	20 (8)	53.5 ± 1.4	0		√	√	√		√	9.6	↓22% *	↓4%	NV	↓1%	[179]
PLP (various)	2776	Diet	Tid	56 ^P^	20 (11)	53.0 ± 1.2	9			√	√	√		7.7	↓15% *	↓5%	↓6%	↓6% *	[180]
PLP (various) + omega-3	2667	Diet	Tid	56 ^P^	19 (11)	55.0 ± 1.2	9			√	√	√		5.4	↓12% *	↓1%	↓5%	↓8% *	[180]
Cranberry (proanthocyanidins, anthocyanidins)	458	Diet (drink)	Bid	56 ^P^	15 (15)	52.0 ± 1.1	3			√	√		√	8.6	↑4%	↓3%	↓4%	↓3%	[181]
Cranberry and strawberries (phenolic acids, pro-anthocyanidins)	333	Liquid supplement	Die	42 ^P^	20 (11)	57.0 ± 1.0	9		√	√	√	√		9.1	↓10%	↓2%	NV	↑1%	[182]
Red wine (catechin, epicatechin, gallic acid)	798	Diet (drink)	Die	28 ^CO^	67 (0)	60.0 ± 1.0	8	√		√		√	√	>30	↑2%	↓1%	↓4%	↑7% *	[183]
Dealcoholized wine (catechin, epicatechin, gallic acid)	733	Diet (drink)	Die	28 ^CO^	67 (0)	60.0 ± 1.0	8	√		√		√	√	>30	↓2%	↓4%	↓2%	NV	[183]
Pomegranate (not specified)	119.1	Diet (drink)	die	365 ^P^	66 (29)	65.9 ± 1.4	34		√	√			√	24.5	↓9% *	↑1%	↑6%	↑11% *	[184]
Onion (quercetin)	162	Capsule	Tid	21 ^CO^	68 (34)	47.4 ± 1.5	3	√	√		√		√	9.3	↑1%	↓1%	↓1%	↓2%	[185]
Quercetin	150	Capsule	Tid	56 ^CO^	19 (0)	59.5 ± 1.4	0				√	√	√	13.3	↑31%	↑5% *	↑3%	↓2% *	[186]
Quercetin	150	Capsule	Tid	56 ^CO^	30 (0)	59.4 ± 0.9	0	√			√	√	√	15.6	↑4%	↑2%	↑2%	↓1% *	[186]
Resveratrol	150	Capsule	Die	30 ^CO^	11 (0)	52.5 ± 2.1	0		√		√			11.2	↑13% *	N/A	N/A	N/A	[187]

^1^ Baseline characteristics of participants include 3 or more of the following parameters in order to be associated with the metabolic syndrome: abdominal obesity (BMI > 30 kg × m^−2^ and/or WC > 102 cm (male)/88 cm (female)), hypertension (SBP > 130 mmHg), insulin resistance presenting as elevated fasting blood glucose (>5.5 mM), hyperTG (>1.7 mM) and low HDL-c (<1 mM (male)/1.3 mM (female)). ^2^ Change as a percentage from baseline. Up and down arrows indicate lipid/lipoprotein increase and decrease, respectively, following PLP challenge. * indicates significant variation (*p* < 0.05). ^3^ As specified by the authors in the case of a non-purified extracts. ^4^ Values represent mean ± standard error of the mean. Bid, twice a day; BMI, body mass index; ^CO^, cross-over intervention; Die, daily; D.-O., drop-out rate; FRS, Framingham risk score; HDL-C, high-density lipoprotein-cholesterol; IR, insulin resistance; LDL-C, low-density lipoprotein-cholesterol; N/A, not available; NV, no variation; ^P^, parallel intervention; PLP, polyphenol; SBP, Systolic blood pressure; S.D., study design TC, total cholesterol; TG, triglycerides; Tid, thrice a day.

**Table 8:** Clinical studies evaluating lipid/lipoprotein status of participants with type 2 diabetes ^1^ in response to polyphenol supplementation.

Polyphenols			Protocol		Participants			Variation of Lipid Profile ^2^				Reference
Dietary Source (Main PLP) ^3^	Dosage (mg/Day)	Matrix	Intake Repartition	Length ^S.D^ (Days)	*n* (Female)	Age ^4^ (Years)	D.-O. (%)	TG	TC	LDL- C	HDL-C	
Black soybean (proanthocyanidin, isoflavone) (+120 mg fenofibrate) (+70 mg fibers)	538	Capsule	Die	56 ^P^	7 (3)	57.4 ± 4.3	N/A	↓42% *	↓6%	↓15% *	↑11%	[188]
Black soybean (proanthocyanidin, isoflavone) (+70 mg fibers)	538	Capsule	Die	56 ^P^	18 (6)	56.7 ± 2.7	N/A	↓13%	↑2%	↓1%	↑2%	[188]
Chlorogenic acid	1200	Capsule	Tid	84 ^P^	14 (14)	43.0 ± 1.7	13	↓19% *	↓4% *	↓17% *	↑6%	[189]
Curcuminoid	70	Capsule	Die	56 ^P^	37 (20)	46.4 ± 1.7	7	↓13% *	↓12% *	↓11%	↑5%	[190]
Grapefruit, green tea, black carrot and guarana seed extract (no information provided)	370	Capsule	Bid	84 ^P^	8 (4)	40.7 ± 0.7	0	↓14% *	↓9% *	↓10% *	↑9% *	[191]
Resveratrol	40	Capsule	Die	183 ^P^	59 (25)	64.9 ± 1.1	7	↑1%	↑5%	↑7%	↑1%	[177]
Resveratrol	500	Capsule	Die	183 ^P^	62 (23)	65.0 ± 0.9	7	↑21% *	↑6% *	↑6%	NV	[177]

^1^ As specified in inclusion criteria of study population or subgroup. ^2^ Change as a percentage of the baseline. Up and down arrows indicate lipid/lipoprotein increase and decrease, respectively, following PLP challenge. * indicates significant variation (*p* < 0.05). ^3^ As specified by the authors in the case of a non-purified extracts. ^4^ Values represent mean ± standard error of the mean. Bid, twice a day; Die, daily; D.-O., drop-out rate; ^CO^, cross-over intervention; HDL-C, high-density lipoprotein-cholesterol; LDL-C, low-density lipoprotein-cholesterol; N/A, not available; NV, no variation; ^P^, parallel intervention; PLP, polyphenol; S.D., study design; TC, total cholesterol; TG, triglycerides; Tid, thrice a day.

### 5.2. Postprandial Interventions

As we postulated that chronic intake of PLPs over an extended period of time could ameliorate lipidemia, we similarly investigated their postprandial potential through acute intake studies. The studies summarized in Table 9 comprised a high-fat challenge, alternatively referred to as an oral lipid tolerance test. This was administered to participants either following a period of chronic intake of PLPs (14–56 days) or alternatively at a single time point with no prior chronic intake. Reduction of TG is the most frequent beneficial outcome reported (↓5–39%) although improvements in TC, LDL-C and HDL-C were noted. Absence or negative variation of lipidemia was reported in 6 studies. These were all acute postprandial challenges without prior chronic intake and were made of either healthy [192,193,194] or DLP participants [195,196]. High-fat meal composition (both in terms of energy density and fat) and intervention length, following ingestion, adds even greater heterogeneity to these trials. This issue originates from the fact that there is no standardized universally accepted oral lipid tolerance test. Indeed, challenges can differ based on fat content (5–140 g), macronutrient composition and time of measurement following ingestion (2–10 h) [197,198,199]. As a reference, an expert panel in 2011 recommended for the sake of standardization and repeatability that an oral lipid tolerance test should consist of an 8-hour fast followed by a high-fat meal comprising 75 g of fat with a single measurement of TG after 4 hours [200].

In all trials, the composition of the high-fat meal displayed moderate amounts of lipids (ranging from 25 to 60 g) with sparse information on fat saturation and additional macronutrients. Interestingly, nearly half of the included studies were closely part of a chronic supplementation protocol, which provides more beneficial effects towards lowering of postprandial TG and OxS. However, it should be noted that participants had either baseline DLP, MetS or T2D background, whereas the remaining trials (with no adjunct chronic intake) generally aimed at investigating healthy participants. Nevertheless, the composition of supplemented PLPs was not reported. However, in some cases, when composition was rigorously described, PLP metabolites such as quercetin dehydrate and resveratrol were found to significantly prevent the rise of postprandial TG or Apo B-48/100 production rates [163,186].

Postprandial trials also tend to address OxS, inflammation, glucose intolerance and IR. In their study, Farràs et al. [192] reported that PLPs from olive oil increased the gene expression of circulating white blood cell biomarkers in association with DLP, OxS and inflammation.

### 5.3. Matrix and Methods of Delivery

Regarding optimal polyphenoclic challenge, there is an extreme heterogeneity in study design and there appears to be a lack of consensus regarding their most favorable administration in human interventions. Indeed, there is a notable absence of studies comparing the preferable matrix for supplementation (i.e., whole food, liquid supplement or purified capsule), dosage or repartition throughout the day (e.g., die, bid, tid, with or without meal, etc.).

Since evidence suggests that the hypolipidemic actions of PLPs may initially come into play in the gut either through nutritional or bile acid chelation, and inhibition of pancreatic lipase, thereby limiting lipid absorption, polyphenolic supplementation should ideally be done in clinical trials during meal intakes for optimal effect such as in the case of other nutritional binders [204]. Nevertheless, this precaution is rarely addressed in clinical trials centered on DLP, where instructions are instead focused on patient adherence rather than on the cum cibum (cc) potential of PLPs. Consequently, polyphenolic intake is usually once to twice a day and is less commonly extended to thrice a day. The only circumstance under which PLPs are systematically administered with food intake is in the case where their source derives from the entire diet regimen. These are the studies that systematically present the greatest benefits on lipid/lipoprotein status.

### 5.4. Dosage

A recurring and puzzling problem in clinical trials is that high polyphenolic doses (e.g., >500 mg/day) are not necessarily associated with a better outcome on lipid profile [177,178,185,188]. A recent review on polyphenolic consumption showed that populations with either a 1170 or 2632 mg/day intake reduced atherosclerosis risk and T2D-related events, respectively [205]. However, mounting evidence points that flavonoids and their subclasses could have a decreasing linear dose-response effect on lipidemia, most notably between the 100–400 mg/day intake [206]. As these conclusions are drawn from prospective studies with whole foods instead of isolated supplements in randomized controlled trials, caution is warranted as the suggested hypolipidemic effects cannot be exclusively attributed to PLPs.

Nevertheless, this suggests that aside from dosage, there may be other factors at play which could mitigate polyphenolic intervention, such as qualitative composition, bioavailability and method of delivery. This complexity is reflected by the lack of solid advice regarding daily intake. As opposed to other nutraceuticals, there presently lacks any form of official recommendation for PLPs in terms of blood lipid management [73]. Our showings demonstrate that no single intake of any given PLP convincingly ameliorates blood lipids. Rather, a combination of PLP, reflective of a more natural, unprocessed intake of foods, appears to be the most important criteria. This highlights the importance for future studies to properly assess not only composition of supplements used, by the bioavailability of PLP as well. For now, the closest official nutritional recommendation available stems from the European Food Safety Authority, which in 2012 certified PLPs from olives and olive-derived products as safe-warranting normal HDL-C blood levels and limiting LDL oxidation [207]. In a similar panel, the European Food Safety Authority also concluded that flavanols from cocoa origin were beneficial to endothelium-dependent vasodilatation and recommended a 200 mg/day intake in order to achieve desirable effects [208]. Concomitantly, the USDA considers anthocyanidins as the most potent antioxidants amongst flavonoids to prevent LDL oxidation, without, however, specifying a particular dose or intake recommendations [79,209].

## 6. Conclusions and Future Perspectives

Evidence-based knowledge has been stated herein regarding the effectiveness and indications of PLP-based phytochemicals. Surprisingly, there is currently a research gap relating to the challenge of PLP on primary DLPs in humans. Regarding DLP as a secondary, cardiometabolic complication, lipid-lowering activity of PLPs has been reported in various clinical studies, which we thoroughly and critically examined and analyzed in order to determine whether PLPs have the potential to treat or ameliorate lipid metabolism. Generally, the majority of clinical investigations showed an advantage in treating hypertriglyceridemia and hypercholesterolemia, whether in healthy participants or subjects with one, two and three disturbed lipid/lipoprotein components, or in MetS, T2D and postprandial DLP in response to PLP intake. Despite these promising findings, the review clearly exhibited an invariable or opposite trend as was the case for resveratrol. In these instances, the low study power and sample size may explain the conflicting data. Additionally, the inconsistency may stem from the PLP type, vast concentration range and intake duration, as well as the whole study design. More particularly, matrix represents a great challenge for PLP studies since the presence of non-polyphenolic constituents in fruits and vegetables may interfere with the pharmacological responses to PLP phytocomplex. The future of collection evidence as to the efficacy of PLPs in preventing or curtailing dyslipidemic risk factors entails the achievement of rigorously clinical trials with a well-defined design, stringent enrollment criteria, optimum dose and well-characterized PLPs formulations along with specific alimentary regimen, anticipated endpoints and extended follow-ups. Only then can we reach the goals of clinical PLP use in DLP without potentially being biased by a number of factors.

## Figures and Tables

**Table 1:** Classification of primary hyperlipoproteinemia.

Type	Name	Molecular Defect	Lipoprotein Elevated	Clinical Features	Incidence
1	Familial Hyperchylomicronemia	LPL, Apo C-II	CM	Juvenile or early adulthood onset; Eruptive xanthomas; Lipemia retinalis; Pancreatitis; Hepatosplenomegaly; Dyspnea; Lymphadenopathy; Neurologic dysfunction	1:1,000,000
2a	Familial Hypercholesterolemia	a. LDLR b. Apo B-100 c. PCSK9	LDL	Onset at all ages; Tendon xanthomas, Arthralgia; Xanthelasmas; Corneal arcus	a. 1:500 b. <1:1000 c. 1:1,000,000
2b	Combined HLP	Polygenetic	LDL VLDL	CVD	1:50–1:200
3	Dysbetalipoproteinemia	Apo E	IDL CM-remnants	Palmar xanthomas; CVD	1:1000–1:5000
4	Primary or simple hypertriglyceridemia	Unknown	VLDL	Adult onset; Eruptive xanthomas; Hepatosplenomegaly; Hyperglycemia; Hyperuricemia	1:50–1:100
5	Mixed hypertriglyceridemia	Unknown	CM VLDL	Eruptive xanthomas; Pancreatitis; CVD	Rare

Adapted from [8,28]. CM, chylomicron; CVD, cardiovascular disease; FH, familial hypercholesterolemia; HLP, hyperlipoproteinemia; IDL, intermediate-density lipoprotein; LDL, low-density lipoprotein; LDLR, low-density lipoprotein receptor; LPL, lipoprotein lipase; PCSK9, proprotein convertase subtilisin/kexin type 9; VLDL, very-low-density lipoprotein.

**Table 3:** Clinical trials evaluating lipid/lipoprotein status of healthy participants in response to PLP supplementation.

Polyphenols			Protocol		Participants			Variation of Lipid Profile ^1^				Reference
Dietary Source (Main PLP) ^2^	Dosage (mg/Day)	Matrix	Intake Repartition	Length ^S.D.^ (Days)	*n* (Female)	Age ^3^ (Years)	D.-O. (%)	TG	TC	LDL-C	HDL-C	
Red grape (anthocyanidins, quercetin, myricetin)	640	Diet (drink)	Bid	14 ^P^	15 (7)	34.4 ± 3.3	10	↑19%	↓6% *	↓13% *	↑16% *	[89]
Potato (anthocyanidins)	288	Diet (whole food)	Die	14 ^CO^	14 (8)	33.5 ± 2.9	0	↓11% *	↑9%	↓11% *	NV	[137]
Shampion apple (quercetin, epicatechin) + pectin	75	Diet (apple pomace)	Die	28 ^CO^	23 (14)	36.2 ± 3.7	32	↓11%	↓5% *	↓10% *	NV	[143]
Shampion apple (Procyanidin, Epicatechin) + pectin	239	Diet (whole apple)	Die	28 ^CO^	23 (14)	36.2 ± 3.7	32	↓7% *	↓7% *	↓8% *	↓6%	[143]
Shampion apple (Procyanidin, chlorogenic acid)	145	Diet (cloudy apple juice)	Die	28 ^CO^	23 (14)	36.2 ± 3.7	32	↑1%	↓3% *	↓4% *	NV	[143]
Shampion apple (Procyanidin, chlorogenic acid)	108	Diet (clear apple juice)	Die	28 ^CO^	23 (14)	36.2 ± 3.7	32	↑4%	↑2% *	↑6% *	↓1%	[143]
Yerba mate tea (green or roasted) (cholorogenic acid, 4,5-dicaffeolquinic acid, gallocatechin)	3589	Diet (drink)	Tid	40 ^P^	15 (14)	42.0 ± 3.2	11	NV	↓3% *	↓7% *	↑2%	[144]
Yerba mate tea (green or roasted) (cholorogenic acid, 4,5-dicaffeolquinic acid, gallocatechin)	3589	Diet (drink)	Tid	20 ^P^	15 (14)	42.0 ± 3.2	11	↑13%	↓2%	↓9% *	↑4%	[144]
Olive leaf extract (oleuropein)	167	Liquid supplement	Bid	42 ^CO^	60 (0)	45.3 ± 1.6	2	↓12% *	↓6% *	↓6% *	↓4%	[145]
Chocolate (flavanol, epicatechin) + fibers	45.3	Diet (drink)	Bid	28 ^CO^	24 (13)	27.0 ± 4.8	12	↓2%	↑4%	↑1%	↑16% *	[146]
Resveratrol	150	Capsule	Die	30 ^CO^	15 (12)	38.2 ± 2.1	18	↓1%	↑2%	↑2%	↑1%	[85]
Resveratrol (+300mg Orlistat die)	300	Capsule	Tid	168 ^P^	24 (21)	40.9 ± 1.6	48	↓7%	N/A	N/A	N/A	[147]
Resveratrol	300	Capsule	Tid	168 ^P^	15 (12)	33.7 ± 2.0	48	↑10%	N/A	N/A	N/A	[147]
Resveratrol	500	Capsule	Die	30 ^CO^	49 (42)	35.9 ± 1.6	0	↓0.4% *	NV	N/A	↓1%	[148]
Coffee (hydroxycinnamic acids, methylxanthines)	510.6	Diet (drink)	Tid	56 ^CO^	25 (15)	26.2 ± 1.4	4	NV	N/A	N/A	↑4%	[149]

^1^ Change as percentage of baseline. Up and down arrows indicate lipid/lipoprotein increase and decrease, respectively, following PLP challenge. * indicates significant variation (*p* < 0.05). ^2^ As specified by the authors in the case of a non-purified extracts. ^3^ Values represent mean ± standard error of the mean. Bid, twice a day; ^CO^, cross-over intervention; Die, daily; D.-O., drop-out rate; HDL-C, high-density lipoprotein cholesterol; LDL-C, low-density lipoprotein cholesterol; N/A, not available; NV, no variation; ^P^, parallel intervention; PLP, polyphenol; S.D., Study design; TC, total cholesterol; TG, triglycerides; Tid, thrice a day.

**Table 9:** Clinical studies evaluating the effect of polyphenols on postprandial dyslipidemia.

Polyphenol			Length of Chronic Intake (Days; if Available) ^1^	Composition of High-Fat Meal		Length of Challenge (Hours)	Participants N (Female); Baseline Characteristics	Reported Effects	Reference
Dietary Source	Dosage (mg)	Matrix		Energy (kcal)	Fat (g)				
Quercetin dihydrates	150	Capsule	56	N/A	60	8	19 (0) MetS, ApoE3 homozygotes	↓11% * of AUC 0–4 h—TG vs placebo; No effect overall on other lipid parameters, glucose and insulin levels.	[186]
Quercetin dihydrates	150	Capsule	56	N/A	60	8	30 (0) MetS, ApoE3/E4 heterozygotes	↓11% * AUC 0–4 h—TG vs placebo; No effect overall on other lipid parameters, glucose and insulin levels.	[186]
Tea, coffee, chocolate, fruits, olive oil	2903	Diet	21	1000	N/A	6	20 (11) MetS	↓39% * AUC 0–6 h—TG vs baseline; ↓39% * AUC 0–6 h—VLDL-TG ↓90% * AUC 0–6 h—VLDL-TC ↓81% * AUC 0–6 h—Apo B-48; No effect on CM composition.	[180]
Resveratrol	1500	Capsule	14	N/A	49%	10	8 (0) DLP	↓22% * Apo B-48 and ↓27% * ApoB100 production rates; No effect on plasma TG, TRL-TG, glucose and insulin levels.	[163]
Strawberry	338	Liquid supplement	42	960	31	6	24 (14) DLP	↓5% * TG, ↓4% * TC, ↓4% * LDL-C ↓3% * HDL-C ↓48% oxLDL vs placebo.	[201]
Strawberry	338	Liquid supplement	0	960	31	6	24 (14) DLP	↓3% * TG; ↑1% * LDL-C; ↓1% * HDL-C; ↓115% * oxLDL vs placebo; No effect on TC.	[201]
Cocoa	960	Liquid supplement	0	766	50	6	18 (14) T2D	↑2% * HDL-C; ↑overall insulin levels * vs placebo; No effect overall on other lipid parameters and glucose levels.	[202]
Red wine (no alcohol)	880	Liquid supplement	0	N/A	25	7	17 (17) DLP	No effect on TG, Apo B-48 and insulin levels vs placebo.	[196]
Olive oil	8	Diet	0	N/A	27	5	13 (6) Healthy	↑15% * TG; ↓9% * oxLDL; ↓7% * glucose vs baseline; no other effect on lipid, OxS or inflammation parameters.	[192]
Olive oil	26	Diet	0	N/A	27	5	13 (6) Healthy	↑24% * TG; ↓7% * oxLDL; ↓6% * glucose vs baseline; no other effect on lipid, OxS or inflammation parameters.	[192]
Pomegranate	652–948	Liquid supplement	0	N/A	50	2	19 (0) Healthy	No effect overall on lipid parameters.	[195]
Red wine	561	Diet	0	N/A	26	3	12 (6) Healthy	↑15% * TG ↓lipid hydroperoxides *, oxyCHOLs *, 7-ketoCHOL * and 7-β-hydroxyCHOL * vs placebo.	[203]
Strawberry	196	Powder	0	N/A	50	4	30 (13) DLP	No effect overall on TG, glucose, insulin and OxS levels.	[193]
Coffee	600	Liquid supplement	0	N/A	30	6	13 (0) Healthy	No effect overall on TG, TC, glucose, insulin, OxS and inflammation levels.	[194]

^1^ If not available, this implies that the reported trial did not include chronic intake. * Indicates significant variation (*p* < 0.05). AUC, area under the curve; DLP, dyslipidemia; HDL-C, high-density lipoprotein-cholesterol; LDL-C, low-density lipoprotein-cholesterol; MetS, metabolic syndrome; N/A, not available; oxLDL, oxidized low-density lipoprotein; OxS, oxidative stress; PLP, polyphenol; TC, total cholesterol; TG, triglycerides; TRL, triglyceride-rich lipoprotein; VLDL-TC, very low-density lipoprotein total cholesterol; VLDL-TG, very low-density lipoprotein triglycerides.

## Data Availability

No new data were created or analyzed in this study. Data sharing is not applicable to this article.

## Abbreviations

Apo	Apolipoprotein
ABCA1	ATP-binding cassette transporter A1
CVD	Cardiovascular disease
CHOL	Cholesterol
CM	Chylomicron
CD-36	Cluster of Differentiation-36
DLP	Dyslipidemia
FH	Familial hypercholesterolemia
FA	Fatty acid
HDL-C	High-density lipoprotein cholesterol
HLP	Hyperlipoproteinemia
IR	Insulin resistance
LCAT	lecithin cholesteryl ester transfer protein
LDL-C	Low-density lipoprotein cholesterol
LDLR	LDL receptor
LPL	Lipoprotein lipase
MetS	Metabolic syndrome
MTTP	Microsomal triglyceride transport protein
NPC1L1	Niemann-Pick-C1-like-1
OxS	Oxidative stress
PLP	Polyphenol
PCSK9	Proprotein convertase subtilisin/kexin type 9
ROS	Reactive oxygen species
SR-B1	Scavenger receptor B-1
TC	Total cholesterol
TG	Triglycerides
T2D	Type 2 diabetes
VLDL	Very-low-density lipoprotein

## References

[1] Roth G.A., Johnson C., Abajobir A., Abd-Allah F., Abera S.F., Abyu G., Ahmed M., Aksut B., Alam T., Alam K. (2017). Global, Regional, and National Burden of Cardiovascular Diseases for 10 Causes, 1990 to 2015. J. Am. Coll. Cardiol..

[2] Fryar C.D., Chen T.C., Li X. (2012). Prevalence of uncontrolled risk factors for cardiovascular disease: United States, 1999–2010. NCHS Data Brief.

[3] Reiner Z., Catapano A.L., De Backer G., Graham I., Taskinen M.R., Wiklund O., Agewall S., Alegria E., Chapman M.J., Durrington P. (2011). ESC/EAS Guidelines for the management of dyslipidaemias: The Task Force for the management of dyslipidaemias of the European Society of Cardiology (ESC) and the European Atherosclerosis Society (EAS). Eur. Heart J..

[4] Rees K., Hartley L., Flowers N., Clarke A., Hooper L., Thorogood M., Stranges S. (2013). ‘Mediterranean’ dietary pattern for the primary prevention of cardiovascular disease. Cochrane Database Syst. Rev..

[5] Kastorini C.M., Milionis H.J., Esposito K., Giugliano D., Goudevenos J.A., Panagiotakos D.B. (2011). The effect of Mediterranean diet on metabolic syndrome and its components: A meta-analysis of 50 studies and 534,906 individuals. J. Am. Coll. Cardiol..

[6] Mehmood A., Usman M., Patil P., Zhao L., Wang C. (2020). A review on management of cardiovascular diseases by olive polyphenols. Food Sci. Nutr..

[7] Hussain M.M. (2014). Intestinal lipid absorption and lipoprotein formation. Curr. Opin. Lipidol..

[8] Semenkovich C.F., Goldman L., Schafer A.I. (2012). Disorders of Lipid Metabolism. Goldman’s Cecil Medicine.

[9] Jacobson T.A., Ito M.K., Maki K.C., Orringer C.E., Bays H.E., Jones P.H., McKenney J.M., Grundy S.M., Gill E.A., Wild R.A. (2015). National lipid association recommendations for patient-centered management of dyslipidemia: Part 1--full report. J. Clin. Lipidol..

[10] Anderson T.J., Grégoire J., Pearson G.J., Barry A.R., Couture P., Dawes M., Francis G.A., Genest J., Grover S., Gupta M. (2016). 2016 Canadian Cardiovascular Society Guidelines for the Management of Dyslipidemia for the Prevention of Cardiovascular Disease in the Adult. Can. J. Cardiol..

[11] Hansson G.K., Hamsten A., Goldman L., Schafer A.I. (2012). Atherosclerosis, Thrombosis, and Vascular Biology. Goldman’s Cecil Medicine.

[12] Levy E., Poinsot P., Spahis S. (2019). Chylomicron retention disease: Genetics, biochemistry, and clinical spectrum. Curr. Opin. Lipidol..

[13] Levy E. (2015). Insights from human congenital disorders of intestinal lipid metabolism. J. Lipid Res..

[14] Ko C.W., Qu J., Black D.D., Tso P. (2020). Regulation of intestinal lipid metabolism: Current concepts and relevance to disease. Nat. Rev. Gastroenterol. Hepatol..

[15] Warnakula S., Hsieh J., Adeli K., Hussain M.M., Tso P., Proctor S.D. (2011). New insights into how the intestine can regulate lipid homeostasis and impact vascular disease: Frontiers for new pharmaceutical therapies to lower cardiovascular disease risk. Can. J. Cardiol..

[16] Hsieh J., Hayashi A.A., Webb J., Adeli K. (2008). Postprandial dyslipidemia in insulin resistance: Mechanisms and role of intestinal insulin sensitivity. Atheroscler. Suppl..

[17] Roy C.C., Levy E., Green P.H., Sniderman A., Letarte J., Buts J.P., Orquin J., Brochu P., Weber A.M., Morin C.L. (1987). Malabsorption, hypocholesterolemia, and fat-filled enterocytes with increased intestinal apoprotein B. Chylomicron retention disease. Gastroenterology.

[18] Jones B., Jones E.L., Bonney S.A., Patel H.N., Mensenkamp A.R., Eichenbaum-Voline S., Rudling M., Myrdal U., Annesi G., Naik S. (2003). Mutations in a Sar1 GTPase of COPII vesicles are associated with lipid absorption disorders. Nat. Genet..

[19] Abumrad N.A., Davidson N.O. (2012). Role of the gut in lipid homeostasis. Physiol. Rev..

[20] Levy E., Roy C.C., Thibault L., Bonin A., Brochu P., Seidman E.G. (1994). Variable expression of familial heterozygous hypobetalipoproteinemia: Transient malabsorption during infancy. J. Lipid Res..

[21] Young S.G., Hubl S.T., Smith R.S., Snyder S.M., Terdiman J.F. (1990). Familial hypobetalipoproteinemia caused by a mutation in the apolipoprotein B gene that results in a truncated species of apolipoprotein B (B-31). A unique mutation that helps to define the portion of the apolipoprotein B molecule required for the formation of buoyant, triglyceride-rich lipoproteins. J. Clin. Investig..

[22] Lee S.J., Grosskopf I., Choi S.Y., Cooper A.D. (2004). Chylomicron remnant uptake in the livers of mice expressing human apolipoproteins E3, E2 (Arg158-->Cys), and E3-Leiden. J. Lipid Res..

[23] Morgantini C., Xiao C., Dash S., Lewis G.F. (2014). Dietary carbohydrates and intestinal lipoprotein production. Curr. Opin. Clin. Nutr. Metab. Care.

[24] Levy E., Spahis S., Ziv E., Marette A., Elchebly M., Lambert M., Delvin E. (2006). Overproduction of intestinal lipoprotein containing apolipoprotein B-48 in Psammomys obesus: Impact of dietary n-3 fatty acids. Diabetologia.

[25] Zoltowska M., Ziv E., Delvin E., Sinnett D., Kalman R., Garofalo C., Seidman E., Levy E. (2003). Cellular aspects of intestinal lipoprotein assembly in Psammomys obesus: A model of insulin resistance and type 2 diabetes. Diabetes.

[26] Haidari M., Leung N., Mahbub F., Uffelman K.D., Kohen-Avramoglu R., Lewis G.F., Adeli K. (2002). Fasting and postprandial overproduction of intestinally derived lipoproteins in an animal model of insulin resistance. Evidence that chronic fructose feeding in the hamster is accompanied by enhanced intestinal de novo lipogenesis and ApoB48-containing lipoprotein overproduction. J. Biol. Chem..

[27] Xiao C., Dash S., Morgantini C., Lewis G.F. (2014). New and emerging regulators of intestinal lipoprotein secretion. Atherosclerosis.

[28] Cox R.A., Garcia-Palmieri M.R., Walker H.K., Hall W.D., Hurst J.W. (1990). Cholesterol, Triglycerides, and Associated Lipoproteins. Clinical Methods: The History, Physical, and Laboratory Examinations.

[29] Gomez-Delgado F., Alcala-Diaz J.F., Leon-Acuña A., Lopez-Moreno J., Delgado-Lista J., Gomez-Marin B., Roncero-Ramos I., Yubero-Serrano E.M., Rangel-Zuñiga O.A., Vals-Delgado C. (2019). Apolipoprotein E genetic variants interact with Mediterranean diet to modulate postprandial hypertriglyceridemia in coronary heart disease patients: CORDIOPREV study. Eur. J. Clin. Invest..

[30] Durst R., Ibe U.K., Shpitzen S., Schurr D., Eliav O., Futema M., Whittall R., Szalat A., Meiner V., Knobler H. (2017). Molecular genetics of familial hypercholesterolemia in Israel-revisited. Atherosclerosis.

[31] Dron J.S., Hegele R.A. (2017). Genetics of Triglycerides and the Risk of Atherosclerosis. Curr. Atheroscler. Rep..

[32] Bjorn L., Trond P.L., Ose L., Hamsten A., Karpe F. (2000). A functional polymorphism in the promoter region of the microsomal triglyceride transfer protein (MTP -493G/T) influences lipoprotein phenotype in familial hypercholesterolemia. Arterioscler. Thromb. Vasc. Biol..

[33] St-Pierre J., Lemieux I., Miller-Felix I., Prud’homme D., Bergeron J., Gaudet D., Nadeau A., Despres J.P., Vohl M.C. (2002). Visceral obesity and hyperinsulinemia modulate the impact of the microsomal triglyceride transfer protein -493G/T polymorphism on plasma lipoprotein levels in men. Atherosclerosis.

[34] Levy E., Spahis S., Garofalo C., Marcil V., Montoudis A., Sinnet D., Sanchez R., Peretti N., Beaulieu J.F., Sane A. (2014). Sar1b transgenic male mice are more susceptible to high-fat diet-induced obesity, insulin insensitivity and intestinal chylomicron overproduction. J. Nutr. Biochem..

[35] Levy E., Spahis S., Sinnett D., Peretti N., Maupas-Schwalm F., Delvin E., Lambert M., Lavoie M.A. (2007). Intestinal cholesterol transport proteins: An update and beyond. Curr. Opin. Lipidol..

[36] Sane A.T., Sinnett D., Delvin E., Bendayan M., Marcil V., Ménard D., Beaulieu J.F., Levy E. (2006). Localization and role of NPC1L1 in cholesterol absorption in human intestine. J. Lipid Res..

[37] Levy E., Ménard D., Suc I., Delvin E., Marcil V., Brissette L., Thibault L., Bendayan M. (2004). Ontogeny, immunolocalisation, distribution and function of SR-BI in the human intestine. J. Cell Sci..

[38] Suc I., Brunet S., Mitchell G., Rivard G.E., Levy E. (2003). Oxidative tyrosylation of high density lipoproteins impairs cholesterol efflux from mouse J774 macrophages: Role of scavenger receptors, classes A and B. J. Cell Sci..

[39] Ulug E., Nergiz-Unal R. (2020). Dietary Fatty Acids and CD36 Mediated Cholesterol Homeostasis: Potential Mechanisms. Nutr. Res. Rev..

[40] Yamamoto H., Yamanashi Y., Takada T., Mu S., Tanaka Y., Komine T., Suzuki H. (2019). Hepatic Expression of Niemann-Pick C1-Like 1, a Cholesterol Reabsorber from Bile, Exacerbates Western Diet-Induced Atherosclerosis in LDL Receptor Mutant Mice. Mol. Pharmacol..

[41] Tomkin G.H. (2010). Dyslipidaemia--hepatic and intestinal cross-talk. Atheroscler. Suppl..

[42] Levy E., Ben Djoudi Ouadda A., Spahis S., Sane A.T., Garofalo C., Grenier É., Emonnot L., Yara S., Couture P., Beaulieu J.-F. (2013). PCSK9 plays a significant role in cholesterol homeostasis and lipid transport in intestinal epithelial cells. Atherosclerosis.

[43] Temel R.E., Brown J.M. (2015). A new model of reverse cholesterol transport: EnTICEing strategies to stimulate intestinal cholesterol excretion. Trends Pharmacol. Sci..

[44] Xie P., Zhu H., Jia L., Ma Y., Tang W., Wang Y., Xue B., Shi H., Yu L. (2014). Genetic demonstration of intestinal NPC1L1 as a major determinant of hepatic cholesterol and blood atherogenic lipoprotein levels. Atherosclerosis.

[45] Yu X.H., Zhang D.W., Zheng X.L., Tang C.K. (2019). Cholesterol transport system: An integrated cholesterol transport model involved in atherosclerosis. Prog. Lipid Res..

[46] Adeli K., Farr J., Xiao S., Lewis C., Gary F., Ridgway N.D., McLeod R.S. (2016). Diabetic Dyslipidaemia. Biochemistry of Lipids, Lipoproteins and Membranes.

[47] Rust S., Rosier M., Funke H., Real J., Amoura Z., Piette J.C., Deleuze J.F., Brewer H.B., Duverger N., Denèfle P. (1999). Tangier disease is caused by mutations in the gene encoding ATP-binding cassette transporter 1. Nat. Genet..

[48] Wellington C.L., Brunham L.R., Zhou S., Singaraja R.R., Visscher H., Gelfer A., Ross C., James E., Liu G., Huber M.T. (2003). Alterations of plasma lipids in mice via adenoviral-mediated hepatic overexpression of human ABCA1. J. Lipid Res..

[49] Basso F., Freeman L., Knapper C.L., Remaley A., Stonik J., Neufeld E.B., Tansey T., Amar M.J., Fruchart-Najib J., Duverger N. (2003). Role of the hepatic ABCA1 transporter in modulating intrahepatic cholesterol and plasma HDL cholesterol concentrations. J. Lipid Res..

[50] Miyazaki O., Fukamachi I., Mori A., Hashimoto H., Kawashiri M.A., Nohara A., Noguchi T., Inazu A., Yamagishi M., Mabuchi H. (2009). Formation of prebeta1-HDL during lipolysis of triglyceride-rich lipoprotein. Biochem. Biophys. Res. Commun..

[51] Barter P.J., Brewer H.B., Chapman M.J., Hennekens C.H., Rader D.J., Tall A.R. (2003). Cholesteryl ester transfer protein: A novel target for raising HDL and inhibiting atherosclerosis. Arterioscler. Thromb. Vasc. Biol..

[52] Yokoyama S. (2006). Assembly of high-density lipoprotein. Arterioscler. Thromb. Vasc. Biol..

[53] Oram J.F. (2003). HDL apolipoproteins and ABCA1: Partners in the removal of excess cellular cholesterol. Arterioscler. Thromb. Vasc. Biol..

[54] Dobiasova M. (2017). Atherogenic impact of lecithin-cholesterol acyltransferase and its relation to cholesterol esterification rate in HDL (FER(HDL)) and AIP [log(TG/HDL-C)] biomarkers: The butterfly effect?. Physiol. Res..

[55] Herscovitz H., Ronen I., Bilu S., Tietz A. (1986). Bile acid synthesis from HDL cholesterol and cholesterol ester by cultured chick embryo hepatocytes. Biochim. Biophys. Acta.

[56] Wanon J., Guertin F., Brunet S., Delvin E., Gavino V., Bouthillier D., Lairon D., Yotov W., Levy E. (1998). The effects of cholesterol uptake from high-density lipoprotein subfractions on biliary sterol secretion in rats with essential fatty-acid deficiency. Hepatology.

[57] Varban M.L., Rinninger F., Wang N., Fairchild-Huntress V., Dunmore J.H., Fang Q., Gosselin M.L., Dixon K.L., Deeds J.D., Acton S.L. (1998). Targeted mutation reveals a central role for SR-BI in hepatic selective uptake of high density lipoprotein cholesterol. Proc. Natl. Acad. Sci. USA.

[58] Fournier M., Bonneil E., Garofalo C., Grimard G., Laverdière C., Krajinovic M., Drouin S., Sinnett D., Marcil V., Levy E. (2019). Altered proteome of high-density lipoproteins from paediatric acute lymphoblastic leukemia survivors. Sci. Rep..

[59] Tall A.R. (2008). Cholesterol efflux pathways and other potential mechanisms involved in the athero-protective effect of high density lipoproteins. J. Intern. Med..

[60] Tall A.R. (2018). Plasma high density lipoproteins: Therapeutic targeting and links to atherogenic inflammation. Atherosclerosis.

[61] Xepapadaki E., Zvintzou E., Kalogeropoulou C., Filou S., Kypreos K.E. (2020). Tauhe Antioxidant Function of HDL in Atherosclerosis. Angiology.

[62] Schmitz G., Drobnik W., Martini L. (2004). ABCA1 Defects. Encyclopedia of Endocrine Diseases.

[63] Inazu A., Brown M.L., Hesler C.B., Agellon L.B., Koizumi J., Takata K., Maruhama Y., Mabuchi H., Tall A.R. (1990). Increased high-density lipoprotein levels caused by a common cholesteryl-ester transfer protein gene mutation. N. Engl. J. Med..

[64] Kopin L., Lowenstein C. (2017). Dyslipidemia. Ann. Intern. Med..

[65] Kockx M., Kritharides L. (2018). Triglyceride-Rich Lipoproteins. Cardiol. Clin..

[66] Sizar O., Khare S., Jamil R.T., Talati R. (2020). Statin Medications.

[67] Goldberg A.C., Hopkins P.N., Toth P.P., Ballantyne C.M., Rader D.J., Robinson J.G., Daniels S.R., Gidding S.S., de Ferranti S.D., Ito M.K. (2011). Familial hypercholesterolemia: Screening, diagnosis and management of pediatric and adult patients: Clinical guidance from the National Lipid Association Expert Panel on Familial Hypercholesterolemia. J. Clin. Lipidol..

[68] Bambauer R., Bambauer C., Lehmann B., Latza R., Schiel R. (2012). LDL-apheresis: Technical and clinical aspects. Sci. World J..

[69] Orringer C.E., Jacobson T.A., Saseen J.J., Brown A.S., Gotto A.M., Ross J.L., Underberg J.A. (2017). Update on the use of PCSK9 inhibitors in adults: Recommendations from an Expert Panel of the National Lipid Association. J. Clin. Lipidol..

[70] Cory H., Passarelli S., Szeto J., Tamez M., Mattei J. (2018). The Role of Polyphenols in Human Health and Food Systems: A Mini-Review. Front. Nutr..

[71] EFSA Panel on Food Additives and Nutrients Sources Added to Food (2015). Risk assessment for peri- and post-menopausal women taking food supplements containing isolated isoflavones. EFSA J..

[72] Trautwein E.A., McKay S. (2020). The Role of Specific Components of a Plant-Based Diet in Management of Dyslipidemia and the Impact on Cardiovascular Risk. Nutrients.

[73] Mach F., Baigent C., Catapano A.L., Koskinas K.C., Casula M., Badimon L., Chapman M.J., De Backer G.G., Delgado V., Ference B.A. (2020). 2019 ESC/EAS Guidelines for the management of dyslipidaemias: Lipid modification to reduce cardiovascular risk. Eur. Heart J..

[74] Jaffe R., Mani J., Watson R.R., Preedy V.R., Zibadi S. (2014). Polyphenolics Evoke Healing Responses: Clinical Evidence and Role of Predictive Biomarkers. Polyphenols in Human Health and Disease.

[75] Cires M.J., Wong X., Carrasco-Pozo C., Gotteland M. (2016). The Gastrointestinal Tract as a Key Target Organ for the Health-Promoting Effects of Dietary Proanthocyanidins. Front. Nutr..

[76] Del Rio D., Rodriguez-Mateos A., Spencer J.P., Tognolini M., Borgesm G., Crozierm A. (2013). Dietary (poly)phenolics in human health: Structures, bioavailability, and evidence of protective effects against chronic diseases. Antioxid. Redox Signal..

[77] Koch W. (2019). Dietary Polyphenols-Important Non-Nutrients in the Prevention of Chronic Noncommunicable Diseases. A Systematic Review. Nutrients.

[78] Manach C., Scalbert A., Morand C., Rémésy C., Jiménez L. (2004). Polyphenols: Food sources and bioavailability. Am. J. Clin. Nutr..

[79] Guo H., Xia M., Watson R.R., Preedy V.R., Zibadi S. (2014). Anthocyanins and Diabetes Regulation. Polyphenols: Mechanisms of Action in Human Health and Disease.

[80] Christensen L.P., Christensen K.B., Watson R.R., Preedy V.R., Zibadi S. (2014). The Role of Direct and Indirect Polyphenolic Antioxidants in Protection Against Oxidative Stress. Polyphenols in Human Health and Disease.

[81] Sies H., Berndt C., Jones D.P. (2017). Oxidative Stress. Annu. Rev. Biochem..

[82] Toyokuni S. (2011). Mysterious link between iron overload and CDKN2A/2B. J. Clin. Biochem. Nutr..

[83] Bast A., Haenen G.R. (2013). Ten misconceptions about antioxidants. Trends Pharmacol. Sci..

[84] Koudoufio M., Desjardins Y., Feldman F., Spahis S., Delvin E., Levy E. (2020). Insight into Polyphenol and Gut Microbiota Crosstalk: Are Their Metabolites the Key to Understand Protective Effects against Metabolic Disorders?. Antioxidants.

[85] Apostolidou C., Adamopoulos K., Iliadis S., Kourtidou-Papadeli C. (2015). Alterations of antioxidant status in asymptomatic hypercholesterolemic individuals after resveratrol intake. Int. J. Food Sci. Nutr..

[86] Naissides M., Mamo J.C., James A.P., Pal S. (2006). The effect of chronic consumption of red wine on cardiovascular disease risk factors in postmenopausal women. Atherosclerosis.

[87] Kroon P.A., Clifford M.N., Crozier A., Day A.J., Donovan J.L., Manach C., Williamson G. (2004). How should we assess the effects of exposure to dietary polyphenols in vitro?. Am. J. Clin. Nutr..

[88] Tsao R. (2010). Chemistry and biochemistry of dietary polyphenols. Nutrients.

[89] Castilla P., Echarri R., Dávalos A., Cerrato F., Ortega H., Teruel J.L., Lucas M.F., Gómez-Coronado D., Ortuño J., Lasunción M.A. (2006). Concentrated red grape juice exerts antioxidant, hypolipidemic, and antiinflammatory effects in both hemodialysis patients and healthy subjects. Am. J. Clin. Nutr..

[90] Furukawa S., Fujita T., Shimabukuro M., Iwaki M., Yamada Y., Nakajima Y., Nakayama O., Makishima M., Matsuda M., Shimomura I. (2004). Increased oxidative stress in obesity and its impact on metabolic syndrome. J. Clin. Investig..

[91] Herranz-Lopez M., Fernández-Arroyo S., Pérez-Sanchez A., Barrajón-Catalán E., Beltrán-Debón R., Menéndez J.A., Alonso-Villaverde C., Segura-Carretero A., Joven J., Micol V. (2012). Synergism of plant-derived polyphenols in adipogenesis: Perspectives and implications. Phytomedicine.

[92] Yeop Han C., Kargi A.Y., Omer M., Chan C.K., Wabitsch M., O’Brien K.D., Wight T.N., Chait A. (2010). Differential effect of saturated and unsaturated free fatty acids on the generation of monocyte adhesion and chemotactic factors by adipocytes: Dissociation of adipocyte hypertrophy from inflammation. Diabetes.

[93] Chung M.Y., Park H.J., Manautou J.E., Koo S.I., Bruno R.S. (2012). Green tea extract protects against nonalcoholic steatohepatitis in ob/ob mice by decreasing oxidative and nitrative stress responses induced by proinflammatory enzymes. J. Nutr. Biochem..

[94] Park H.J., DiNatale D.A., Chung M.Y., Park Y.K., Lee J.Y., Koo S.I., O’Connor M., Manautou J.E., Bruno R.S. (2011). Green tea extract attenuates hepatic steatosis by decreasing adipose lipogenesis and enhancing hepatic antioxidant defenses in ob/ob mice. J. Nutr. Biochem..

[95] Lu C., Zhu W., Shen C.L., Gao W. (2012). Green tea polyphenols reduce body weight in rats by modulating obesity-related genes. PLoS ONE.

[96] Franco J.G., Lisboa P.C., Lima N.S., Amaral T.A., Peixoto-Silva N., Resende A.C., Oliveira E., Passos M.C., Moura E.G. (2013). Resveratrol attenuates oxidative stress and prevents steatosis and hypertension in obese rats programmed by early weaning. J. Nutr. Biochem..

[97] Gomez-Zorita S., Fernández-Quintela A., Macarulla M.T., Aguirre L., Hijona E., Bujanda L., Milagro F., Martínez J.A., Portillo M.P. (2012). Resveratrol attenuates steatosis in obese Zucker rats by decreasing fatty acid availability and reducing oxidative stress. Br. J. Nutr..

[98] Roberts C.K., Sindhu K.K. (2009). Oxidative stress and metabolic syndrome. Life Sci..

[99] Otani H. (2011). Oxidative stress as pathogenesis of cardiovascular risk associated with metabolic syndrome. Antioxid. Redox. Signal..

[100] Bujanda L., Hijona E., Larzabal M., Beraza M., Aldazabal P., García-Urkia N., Sarasqueta C., Cosme A., Irastorza B., González A. (2008). Resveratrol inhibits nonalcoholic fatty liver disease in rats. BMC Gastroenterol..

[101] Li H., Xia N., Forstermann U. (2012). Cardiovascular effects and molecular targets of resveratrol. Nitric Oxide.

[102] Li X.N., Ma L.Y., Ji H., Qin Y.H., Jin S.S., Xu L.X. (2018). Resveratrol protects against oxidative stress by activating the Keap-1/Nrf2 antioxidant defense system in obese-asthmatic rats. Exp. Ther. Med..

[103] Ding H., Heng B., He W., Shi L., Lai C., Xiao L., Ren H., Mo S., Su Z. (2016). Chronic reactive oxygen species exposure inhibits glucose uptake and causes insulin resistance in C2C12 myotubes. Biochem. Biophys. Res. Commun..

[104] Gong L., Guo S., Zou Z. (2020). Resveratrol ameliorates metabolic disorders and insulin resistance in high-fat diet-fed mice. Life Sci..

[105] Brasnyo P., Molnár G.A., Mohás M., Markó L., Laczy B., Cseh J., Mikolás E., Szijártó I.A., Mérei A., Halmai R. (2011). Resveratrol improves insulin sensitivity, reduces oxidative stress and activates the Akt pathway in type 2 diabetic patients. Br. J. Nutr..

[106] Do G.M., Jung U.J., Park H.J., Kwon E.Y., Jeon S.M., McGregor R.A., Choi M.S. (2012). Resveratrol ameliorates diabetes-related metabolic changes via activation of AMP-activated protein kinase and its downstream targets in db/db mice. Mol. Nutr. Food Res..

[107] Su H.C., Hung L.M., Chen J.K. (2006). Resveratrol, a red wine antioxidant, possesses an insulin-like effect in streptozotocin-induced diabetic rats. Am. J. Physiol. Endocrinol. Metab..

[108] Bhatt S.R., Lokhandwala M.F., Banday A.A. (2011). Resveratrol prevents endothelial nitric oxide synthase uncoupling and attenuates development of hypertension in spontaneously hypertensive rats. Eur. J. Pharmacol..

[109] Li X., Dai Y., Yan S., Shi Y., Li J., Liu J., Cha L., Mu J. (2016). Resveratrol lowers blood pressure in spontaneously hypertensive rats via calcium-dependent endothelial NO production. Clin. Exp. Hypertens..

[110] Da Silva Pereira R., Tatsch E., Bochi G.V., Kober H., Duarte T., dos Santos Montagner G.F., da Silva J.E., Duarte M.M., da Cruz I.B., Moresco R.N. (2013). Assessment of oxidative, inflammatory, and fibrinolytic biomarkers and DNA strand breakage in hypercholesterolemia. Inflammation.

[111] Nourooz-Zadeh J., Smith C.C.T., Betteridge D.J. (2001). Measures of oxidative stress in heterozygous familial hypercholesterolaemia. Atherosclerosis.

[112] Tangvarasittichai S. (2015). Oxidative stress, insulin resistance, dyslipidemia and type 2 diabetes mellitus. World J. Diabetes.

[113] Harangi M., Remenyik E.E., Seres I., Varga Z., Katona E., Paragh G. (2002). Determination of DNA damage induced by oxidative stress in hyperlipidemic patients. Mutat. Res..

[114] Khatana C., Saini N.K., Chakrabarti S., Saini V., Sharma A., Saini R.V., Saini A.K. (2020). Mechanistic Insights into the Oxidized Low-Density Lipoprotein-Induced Atherosclerosis. Oxid. Med. Cell. Longev..

[115] Hauck A.K., Bernlohr D.A. (2016). Oxidative stress and lipotoxicity. J. Lipid Res..

[116] Narverud I., Halvorsen B., Nenseter M.S., Retterstøl K., Yndestad A., Dahl T.B., Ulven S.M., Olstad O.K., Ose L., Holven K.B. (2013). Oxidized LDL level is related to gene expression of tumour necrosis factor super family members in children and young adults with familial hypercholesterolaemia. J. Intern. Med..

[117] Holvoet P., Mertens A., Verhamme P., Bogaerts K., Beyens G., Verhaeghe R., Collen D., Muls E., Van de Werf F. (2001). Circulating oxidized LDL is a useful marker for identifying patients with coronary artery disease. Arterioscler. Thromb. Vasc. Biol..

[118] Yang Y.S., Su Y.F., Yang H.W., Lee Y.H., Chou J.I., Ueng K.C. (2014). Lipid-lowering effects of curcumin in patients with metabolic syndrome: A randomized, double-blind, placebo-controlled trial. Phytother. Res..

[119] Fan C., Wo X., Qian Y., Yin J., Gao L. (2006). Effect of curcumin on the expression of LDL receptor in mouse macrophages. J. Ethnopharmacol..

[120] Medzhitov R. (2008). Origin and physiological roles of inflammation. Nature.

[121] Strowig T., Henao-Mejia J., Elinav E., Flavell R. (2012). Inflammasomes in health and disease. Nature.

[122] Curti M.L., Jacob P., Borges M.C., Rogero M.M., Ferreira S.R. (2011). Studies of gene variants related to inflammation, oxidative stress, dyslipidemia, and obesity: Implications for a nutrigenetic approach. J. Obes..

[123] Haybar H., Shahrabi S., Rezaeeyan H., Shirzad R., Saki N. (2019). Endothelial Cells: From Dysfunction Mechanism to Pharmacological Effect in Cardiovascular Disease. Cardiovasc. Toxicol..

[124] Steven S., Frenis K., Oelze M., Kalinovic S., Kuntic M., Bayo Jimenez M.T., Vujacic-Mirski K., Helmstaedter J., Kroeller-Schoen S., Munzel T. (2019). Vascular Inflammation and Oxidative Stress: Major Triggers for Cardiovascular Disease. Oxid. Med. Cell. Longev..

[125] Yuan T., Yang T., Chen H., Fu D., Hu Y., Wang J., Yuan Q., Yu H., Xu W., Xie X. (2019). New insights into oxidative stress and inflammation during diabetes mellitus-accelerated atherosclerosis. Redox Biol..

[126] Bondia-Pons I., Ryan L., Martinez J.A. (2012). Oxidative stress and inflammation interactions in human obesity. J. Physiol. Biochem..

[127] Staels B. (2002). Cardiovascular biology: A cholesterol tether. Nature.

[128] Ansell B.J., Fonarow G.C., Fogelman A.M. (2007). The paradox of dysfunctional high-density lipoprotein. Curr. Opin. Lipidol..

[129] Navab M., Hama S.Y., Hough G.P., Subbanagounder G., Reddy S.T., Fogelman A.M. (2001). A cell-free assay for detecting HDL that is dysfunctional in preventing the formation of or inactivating oxidized phospholipids. J. Lipid Res..

[130] Mitjavila M.T., Moreno J.J. (2012). The effects of polyphenols on oxidative stress and the arachidonic acid cascade. Implications for the prevention/treatment of high prevalence diseases. Biochem. Pharmacol..

[131] Hussain T., Tan B., Yin Y., Blachier F., Tossou M.C., Rahu N. (2016). Oxidative Stress and Inflammation: What Polyphenols Can Do for Us?. Oxid. Med. Cell. Longev..

[132] Vane J.R., Botting R.M. (1998). Mechanism of Action of Nonsteroidal Anti-inflammatory Drugs. Am. J. Med..

[133] Tremaroli V., Backhed F. (2012). Functional interactions between the gut microbiota and host metabolism. Nature.

[134] De Angelis M., Garruti G., Minervini F., Bonfrate L., Portincasa P., Gobbetti M. (2019). The Food-gut Human Axis: The Effects of Diet on Gut Microbiota and Metabolome. Curr. Med. Chem..

[135] Guo X., Tang R., Yang S., Lu Y., Luo J., Liu Z. (2018). Rutin and Its Combination With Inulin Attenuate Gut Dysbiosis, the Inflammatory Status and Endoplasmic Reticulum Stress in Paneth Cells of Obese Mice Induced by High-Fat Diet. Front. Microbiol..

[136] González-Sarrías A., Combet E., Pinto P., Mena P., Dall’Asta M., Garcia-Aloy M., Rodríguez-Mateos A., Gibney E.R., Dumont J., Massaro M. (2017). A Systematic Review and Meta-Analysis of the Effects of Flavanol-Containing Tea, Cocoa and Apple Products on Body Composition and Blood Lipids: Exploring the Factors Responsible for Variability in Their Efficacy. Nutrients.

[137] Tsang C., Smail N.F., Almoosawi S., McDougall G.J.M., Al-Dujaili E.A.S. (2018). Antioxidant Rich Potato Improves Arterial Stiffness in Healthy Adults. Plant Foods Hum. Nutr..

[138] Huang G., Xu J., Guo T.L., Watson R.R., Preedy V.R., Zibadi S. (2018). Exposure to Polyphenolic Compounds Modulates Type 1 Diabetes: The Case of Genistein. Polyphenols: Mechanisms of Action in Human Health and Disease.

[139] Pathak S., Kesavan P., Banerjee A., Banerjee A., Sagdicoglu Celep G., Bissi L., Marotta F., Watson R.R., Preedy V.R., Zibadi S. (2018). Metabolism of Dietary Polyphenols by Human Gut Microbiota and Their Health Benefits. Polyphenols: Mechanisms of Action in Human Health and Disease.

[140] Zhang L., Carmody R.N., Kalariya H.M., Duran R.M., Moskal K., Poulev A., Kuhn P., Tveter K.M., Turnbaugh P.J., Raskin I. (2018). Grape proanthocyanidin-induced intestinal bloom of Akkermansia muciniphila is dependent on its baseline abundance and precedes activation of host genes related to metabolic health. J. Nutr. Biochem..

[141] Roopchand D.E., Carmody R.N., Kuhn P., Moskal K., Rojas-Silva P., Turnbaugh P.J., Raskin I. (2015). Dietary Polyphenols Promote Growth of the Gut Bacterium Akkermansia muciniphila and Attenuate High-Fat Diet-Induced Metabolic Syndrome. Diabetes.

[142] Neyrinck A.M., Van Hée V.F., Bindels L.B., De Backer F., Cani P.D., Delzenne N.M. (2013). Polyphenol-rich extract of pomegranate peel alleviates tissue inflammation and hypercholesterolaemia in high-fat diet-induced obese mice: Potential implication of the gut microbiota. Br. J. Nutr..

[143] Ravn-Haren G., Dragsted L.O., Buch-Andersen T., Jensen E.N., Jensen R.I., Németh-Balogh M., Paulovicsová B., Bergström A., Wilcks A., Licht T.R. (2013). Intake of whole apples or clear apple juice has contrasting effects on plasma lipids in healthy volunteers. Eur. J. Nutr..

[144] De Morais E.C., Stefanuto A., Klein G.A., Boaventura B.C., de Andrade F., Wazlawik E., Di Pietro P.F., Maraschin M., da Silva E.L. (2009). Consumption of yerba mate ( Ilex paraguariensis ) improves serum lipid parameters in healthy dyslipidemic subjects and provides an additional LDL-cholesterol reduction in individuals on statin therapy. J. Agric. Food. Chem..

[145] Lockyer S., Rowland I., Spencer J.P.E., Yaqoob P., Stonehouse W. (2017). Impact of phenolic-rich olive leaf extract on blood pressure, plasma lipids and inflammatory markers: A randomised controlled trial. Eur. J. Nutr..

[146] Martinez-Lopez S., Sarriá B., Sierra-Cinos J.L., Goya L., Mateos R., Bravo L. (2014). Realistic intake of a flavanol-rich soluble cocoa product increases HDL-cholesterol without inducing anthropometric changes in healthy and moderately hypercholesterolemic subjects. Food Funct..

[147] Arzola-Paniagua M.A., García-Salgado López E.R., Calvo-Vargas C.G., Guevara-Cruz M. (2016). Efficacy of an orlistat-resveratrol combination for weight loss in subjects with obesity: A randomized controlled trial. Obesity.

[148] Bo S., Ciccone G., Castiglione A., Gambino R., De Michieli F., Villois P., Durazzo M., Cavallo-Perin P., Cassader M. (2013). Anti-inflammatory and antioxidant effects of resveratrol in healthy smokers a randomized, double-blind, placebo-controlled, cross-over trial. Curr. Med. Chem..

[149] Sarria B., Martínez-López S., Sierra-Cinos J.L., García-Diz L., Mateos R., Bravo-Clemente L. (2018). Regularly consuming a green/roasted coffee blend reduces the risk of metabolic syndrome. Eur. J. Nutr..

[150] Morel S., Leahy J., Fournier M., Lamarche B., Garofalo C., Grimard G., Poulain F., Delvin E., Laverdière C., Krajinovic M. (2017). Lipid and lipoprotein abnormalities in acute lymphoblastic leukemia survivors. J. Lipid Res..

[151] Zhang C., Yuan W., Fang J., Wang W., He P., Lei J., Wang C. (2016). Efficacy of Resveratrol Supplementation against Non-Alcoholic Fatty Liver Disease: A Meta-Analysis of Placebo-Controlled Clinical Trials. PLoS ONE.

[152] Iannelli P., Zarrilli V., Varricchio E., Tramontano D., Mancini F.P. (2007). The dietary antioxidant resveratrol affects redox changes of PPARalpha activity. Nutr. Metab. Cardiovasc. Dis..

[153] Zhang H., Zhang J., Ungvari Z., Zhang C. (2009). Resveratrol improves endothelial function: Role of TNF{alpha} and vascular oxidative stress. Arterioscler. Thromb. Vasc. Biol..

[154] Shen M.Y., Hsiao G., Liu C.L., Fong T.H., Lin K.H., Chou D.S., Sheu J.R. (2007). Inhibitory mechanisms of resveratrol in platelet activation: Pivotal roles of p38 MAPK and NO/cyclic GMP. Br. J. Haematol..

[155] Rivera L., Morón R., Zarzuelo A., Galisteo M. (2009). Long-term resveratrol administration reduces metabolic disturbances and lowers blood pressure in obese Zucker rats. Biochem. Pharmacol..

[156] Fernandez-Castillejo S., Valls R.M., Castañer O., Rubió L., Catalán Ú., Pedret A., Macià A., Sampson M.L., Covas M.I., Fitó M. (2016). Polyphenol rich olive oils improve lipoprotein particle atherogenic ratios and subclasses profile: A randomized, crossover, controlled trial. Mol. Nutr. Food. Res..

[157] Covas M.I., Nyyssönen K., Poulsen H.E., Kaikkonen J., Zunft H.J., Kiesewetter H., Gaddi A., de la Torre R., Mursu J., Bäumler H. (2006). The effect of polyphenols in olive oil on heart disease risk factors: A randomized trial. Ann. Intern. Med..

[158] Devaraj S., Vega-López S., Kaul N., Schönlau F., Rohdewald P., Jialal I. (2002). Supplementation with a pine bark extract rich in polyphenols increases plasma antioxidant capacity and alters the plasma lipoprotein profile. Lipids.

[159] Baba S., Natsume M., Yasuda A., Nakamura Y., Tamura T., Osakabe N., Kanegae M., Kondo K. (2007). Plasma LDL and HDL cholesterol and oxidized LDL concentrations are altered in normo- and hypercholesterolemic humans after intake of different levels of cocoa powder. J. Nutr..

[160] Souza S.J., Petrilli A.A., Teixeira A.M., Pontilho P.M., Carioca A.A., Luzia L.A., Souza J.M., Damasceno N.R., Segurado A.A., Rondó P.H. (2017). Effect of chocolate and mate tea on the lipid profile of individuals with HIV/AIDS on antiretroviral therapy: A clinical trial. Nutrition.

[161] Ruiz-Roso B., Quintela J.C., de la Fuente E., Haya J., Pérez-Olleros L. (2010). Insoluble carob fiber rich in polyphenols lowers total and LDL cholesterol in hypercholesterolemic sujects. Plant Foods Hum. Nutr..

[162] Trautwein E.A., Du Y., Meynen E., Yan X., Wen Y., Wang H., Molhuizen H.O. (2010). Purified black tea theaflavins and theaflavins/catechin supplements did not affect serum lipids in healthy individuals with mildly to moderately elevated cholesterol concentrations. Eur. J. Nutr..

[163] Dash S., Xiao C., Morgantini C., Szeto L., Lewis G.F. (2013). High-dose resveratrol treatment for 2 weeks inhibits intestinal and hepatic lipoprotein production in overweight/obese men. Arterioscler. Thromb. Vasc. Biol..

[164] Novotny J.A., Baer D.J., Khoo C., Gebauer S.K., Charron C.S. (2015). Cranberry juice consumption lowers markers of cardiometabolic risk, including blood pressure and circulating C-reactive protein, triglyceride, and glucose concentrations in adults. J. Nutr..

[165] Gliozzi M., Walker R., Muscoli S., Vitale C., Gratteri S., Carresi C., Musolino V., Russo V., Janda E., Ragusa S. (2013). Bergamot polyphenolic fraction enhances rosuvastatin-induced effect on LDL-cholesterol, LOX-1 expression and protein kinase B phosphorylation in patients with hyperlipidemia. Int. J. Cardiol..

[166] Upadya H., Prabhu S., Prasad A., Subramanian D., Gupta S., Goel A. (2019). A randomized, double blind, placebo controlled, multicenter clinical trial to assess the efficacy and safety of Emblica officinalis extract in patients with dyslipidemia. BMC Complement. Altern. Med..

[167] Kardum N., Milovanović B., Šavikin K., Zdunić G., Mutavdžin S., Gligorijević T., Spasić S. (2015). Beneficial Effects of Polyphenol-Rich Chokeberry Juice Consumption on Blood Pressure Level and Lipid Status in Hypertensive Subjects. J. Med. Food.

[168] Rahbar A.R., Mahmoudabadi M.M., Islam M.S. (2015). Comparative effects of red and white grapes on oxidative markers and lipidemic parameters in adult hypercholesterolemic humans. Food Funct..

[169] Mansur A.P., Roggerio A., Goes M.F.S., Avakian S.D., Leal D.P., Maranhão R.C., Strunz C.M.C. (2017). Serum concentrations and gene expression of sirtuin 1 in healthy and slightly overweight subjects after caloric restriction or resveratrol supplementation: A randomized trial. Int. J. Cardiol..

[170] Chachay V.S., Macdonald G.A., Martin J.H., Whitehead J.P., O’Moore-Sullivan T.M., Lee P., Franklin M., Klein K., Taylor P.J., Ferguson M. (2014). Resveratrol does not benefit patients with nonalcoholic fatty liver disease. Clin. Gastroenterol. Hepatol..

[171] Morissette A., Kropp C., Songpadith J.P., Junges Moreira R., Costa J., Mariné-Casadó R., Pilon G., Varin T.V., Dudonné S., Boutekrabt L. (2020). Blueberry proanthocyanidins and anthocyanins improve metabolic health through a gut microbiota-dependent mechanism in diet-induced obese mice. Am. J. Physiol. Endocrinol. Metab..

[172] Anhe F.F., Varin T.V., Le Barz M., Desjardins Y., Levy E., Roy D., Marette A. (2015). Gut Microbiota Dysbiosis in Obesity-Linked Metabolic Diseases and Prebiotic Potential of Polyphenol-Rich Extracts. Curr. Obes. Rep..

[173] Anhe F.F., Roy D., Pilon G., Dudonné S., Matamoros S., Varin T.V., Garofalo C., Moine Q., Desjardins Y., Levy E. (2015). A polyphenol-rich cranberry extract protects from diet-induced obesity, insulin resistance and intestinal inflammation in association with increased Akkermansia spp. population in the gut microbiota of mice. Gut.

[174] Anhe F.F., Nachbar R.T., Varin T.V., Vilela V., Dudonné S., Pilon G., Fournier M., Lecours M.A., Desjardins Y., Roy D. (2017). A polyphenol-rich cranberry extract reverses insulin resistance and hepatic steatosis independently of body weight loss. Mol. Metab..

[175] Vidal R., Hernandez-Vallejo S., Pauquai T., Texier O., Rousset M., Chambaz J., Demignot S., Lacorte J.M. (2005). Apple procyanidins decrease cholesterol esterification and lipoprotein secretion in Caco-2/TC7 enterocytes. J. Lipid Res..

[176] Yasuda A., Natsume M., Osakabe N., Kawahata K., Koga J. (2011). Cacao polyphenols influence the regulation of apolipoprotein in HepG2 and Caco2 cells. J. Agric. Food. Chem..

[177] Bo S., Ponzo V., Ciccone G., Evangelista A., Saba F., Goitre I., Procopio M., Pagano G.F., Cassader M., Gambino R. (2016). Six months of resveratrol supplementation has no measurable effect in type 2 diabetic patients. A randomized, double blind, placebo-controlled trial. Pharmacol. Res..

[178] Shin H.C., Kim S.H., Park Y., Lee B.H., Hwang H.J. (2012). Effects of 12-week oral supplementation of Ecklonia cava polyphenols on anthropometric and blood lipid parameters in overweight Korean individuals: A double-blind randomized clinical trial. Phytother. Res..

[179] Millar C.L., Duclos Q., Garcia C., Norris G.H., Lemos B.S., DiMarco D.M., Fernandez M.L., Blesso C.N. (2018). Effects of Freeze-Dried Grape Powder on High-Density Lipoprotein Function in Adults with Metabolic Syndrome: A Randomized Controlled Pilot Study. Metab. Syndr. Relat. Disord..

[180] Annuzzi G., Bozzetto L., Costabile G., Giacco R., Mangione A., Anniballi G., Vitale M., Vetrani C., Cipriano P., Della Corte G. (2014). Diets naturally rich in polyphenols improve fasting and postprandial dyslipidemia and reduce oxidative stress: A randomized controlled trial. Am. J. Clin. Nutr..

[181] Basu A., Betts N.M., Ortiz J., Simmons B., Wu M., Lyons T.J. (2011). Low-energy cranberry juice decreases lipid oxidation and increases plasma antioxidant capacity in women with metabolic syndrome. Nutr. Res..

[182] Paquette M., Medina Larqué A.S., Weisnagel S.J., Desjardins Y., Marois J., Pilon G., Dudonné S., Marette A., Jacques H. (2017). Strawberry and cranberry polyphenols improve insulin sensitivity in insulin-resistant, non-diabetic adults: A parallel, double-blind, controlled and randomised clinical trial. Br. J. Nutr..

[183] Chiva-Blanch G., Urpi-Sarda M., Ros E., Valderas-Martinez P., Casas R., Arranz S., Guillén M., Lamuela-Raventós R.M., Llorach R., Andres-Lacueva C. (2013). Effects of red wine polyphenols and alcohol on glucose metabolism and the lipid profile: A randomized clinical trial. Clin. Nutr..

[184] Shema-Didi L., Kristal B., Sela S., Geron R., Ore L. (2014). Does Pomegranate intake attenuate cardiovascular risk factors in hemodialysis patients?. Nutr. J..

[185] Brull V., Burak C., Stoffel-Wagner B., Wolffram S., Nickenig G., Müller C., Langguth P., Alteheld B., Fimmers R., Naaf S. (2015). Effects of a quercetin-rich onion skin extract on 24 h ambulatory blood pressure and endothelial function in overweight-to-obese patients with (pre-)hypertension: A randomised double-blinded placebo-controlled cross-over trial. Br. J. Nutr..

[186] Pfeuffer M., Auinger A., Bley U., Kraus-Stojanowic I., Laue C., Winkler P., Rüfer C.E., Frank J., Bösch-Saadatmandi C., Rimbach G. (2013). Effect of quercetin on traits of the metabolic syndrome, endothelial function and inflammation in men with different APOE isoforms. Nutr. Metab. Cardiovasc. Dis..

[187] Timmers S., Konings E., Bilet L., Houtkooper R.H., van de Weijer T., Goossens G.H., Hoeks J., van der Krieken S., Ryu D., Kersten S. (2011). Calorie restriction-like effects of 30 days of resveratrol supplementation on energy metabolism and metabolic profile in obese humans. Cell Metab..

[188] Kusunoki M., Sato D., Tsutsumi K., Tsutsui H., Nakamura T., Oshida Y. (2015). Black soybean extract improves lipid profiles in fenofibrate-treated type 2 diabetics with postprandial hyperlipidemia. J. Med. Food.

[189] Zuniga L.Y., Aceves-de la Mora M.C.A., González-Ortiz M., Ramos-Núñez J.L., Martínez-Abundis E. (2018). Effect of Chlorogenic Acid Administration on Glycemic Control, Insulin Secretion, and Insulin Sensitivity in Patients with Impaired Glucose Tolerance. J. Med. Food.

[190] Rahmani S., Asgary S., Askari G., Keshvari M., Hatamipour M., Feizi A., Sahebkar A. (2016). Treatment of Non-alcoholic Fatty Liver Disease with Curcumin: A Randomized Placebo-controlled Trial. Phytother. Res..

[191] Cases J., Romain C., Dallas C., Gerbi A., Cloarec M. (2015). Regular consumption of Fiit-ns, a polyphenol extract from fruit and vegetables frequently consumed within the Mediterranean diet, improves metabolic ageing of obese volunteers: A randomized, double-blind, parallel trial. Int. J. Food Sci. Nutr..

[192] Farras M., Valls R.M., Fernández-Castillejo S., Giralt M., Solà R., Subirana I., Motilva M.J., Konstantinidou V., Covas M.I., Fitó M. (2013). Olive oil polyphenols enhance the expression of cholesterol efflux related genes in vivo in humans. A randomized controlled trial. J. Nutr. Biochem..

[193] Richter C.K., Skulas-Ray A.C., Gaugler T.L., Lambert J.D., Proctor D.N., Kris-Etherton P.M. (2017). Incorporating freeze-dried strawberry powder into a high-fat meal does not alter postprandial vascular function or blood markers of cardiovascular disease risk: A randomized controlled trial. Am. J. Clin. Nutr..

[194] Ochiai R., Sugiura Y., Otsuka K., Katsuragi Y., Hashiguchi T. (2015). Coffee bean polyphenols ameliorate postprandial endothelial dysfunction in healthy male adults. Int. J. Food Sci. Nutr..

[195] Mathew A.S., Capel-Williams G.M., Berry S.E., Hall W.L. (2012). Acute effects of pomegranate extract on postprandial lipaemia, vascular function and blood pressure. Plant Foods Hum. Nutr..

[196] Naissides M., Mamo J.C., James A.P., Pal S. (2004). The effect of acute red wine polyphenol consumption on postprandial lipaemia in postmenopausal women. Atherosclerosis.

[197] Guerci B., Paul J.L., Hadjadj S., Durlach V., Vergès B., Attia N., Girard-Globa A., Drouin P. (2001). Analysis of the postprandial lipid metabolism: Use of a 3-point test. Diabetes Metab..

[198] O’Doherty A.F., Sathyapalan T., Rigby A.S., Ingle L., Carroll S. (2018). The repeatability of the abbreviated (4-h) Oral Fat Tolerance Test and influence of prior acute aerobic exercise. Eur. J. Nutr..

[199] Harbis A., Perdreau S., Vincent-Baudry S., Charbonnier M., Bernard M.C., Raccah D., Senft M., Lorec A.M., Defoort C., Portugal H. (2004). Glycemic and insulinemic meal responses modulate postprandial hepatic and intestinal lipoprotein accumulation in obese, insulin-resistant subjects. Am. J. Clin. Nutr..

[200] Kolovou G.D., Mikhailidis D.P., Kovar J., Lairon D., Nordestgaard B.G., Ooi T.C., Perez-Martinez P., Bilianou H., Anagnostopoulou K., Panotopoulos G. (2011). Assessment and clinical relevance of non-fasting and postprandial triglycerides: An expert panel statement. Curr. Vasc. Pharmacol..

[201] Burton-Freeman B., Linares A., Hyson D., Kappagoda T. (2010). Strawberry Modulates LDL Oxidation and Postprandial Lipemia in Response to High-Fat Meal in Overweight Hyperlipidemic Men and Women. J. Am. Coll. Nutr..

[202] Basu A., Betts N.M., Leyva M.J., Fu D., Aston C.E., Lyons T.J. (2015). Acute Cocoa Supplementation Increases Postprandial HDL Cholesterol and Insulin in Obese Adults with Type 2 Diabetes after Consumption of a High-Fat Breakfast. J. Nutr..

[203] Natella F., Macone A., Ramberti A., Forte M., Mattivi F., Matarese R.M., Scaccini C. (2011). Red wine prevents the postprandial increase in plasma cholesterol oxidation products: A pilot study. Br. J. Nutr..

[204] Hutchison A.J. (2009). Oral phosphate binders. Kidney Int..

[205] Del Bo C., Bernardi S., Marino M., Porrini M., Tucci M., Guglielmetti S., Cherubini A., Carrieri B., Kirkup B., Kroon P. (2019). Systematic Review on Polyphenol Intake and Health Outcomes: Is there Sufficient Evidence to Define a Health-Promoting Polyphenol-Rich Dietary Pattern?. Nutrients.

[206] Grosso G., Micek A., Godos J., Pajak A., Sciacca S., Galvano F., Giovannucci E.L. (2017). Dietary Flavonoid and Lignan Intake and Mortality in Prospective Cohort Studies: Systematic Review and Dose-Response Meta-Analysis. Am. J. Epidemiol..

[207] EFSA Panel on Dietetic Products, Nutrition and Allergies (NDA) (2012). Scientific Opinion on the substantiation of a health claim related to polyphenols in olive and maintenance of normal blood HDL cholesterol concentrations (ID 1639, further assessment) pursuant to Article 13(1) of Regulation (EC) No 1924/2006. EFSA J..

[208] EFSA Panel on Dietetic Products, Nutrition and Allergies (NDA) (2012). Scientific Opinion on the substantiation of a health claim related to cocoa flavanols and maintenance of normal endothelium-dependent vasodilation pursuant to Article 13(5) of Regulation (EC) No 1924/2006. EFSA J..

[209] Bhagwat S., Haytowitz D.B., Holden J.M. (2013). USDA Database for the Flavonoid Content of Selected Foods. https://www.ars.usda.gov/arsuserfiles/80400525/data/flav/flav_r03-1.pdf.

